# Comprehensive Functional Analysis of the Enterococcus faecalis Core Genome Using an Ordered, Sequence-Defined Collection of Insertional Mutations in Strain OG1RF

**DOI:** 10.1128/mSystems.00062-18

**Published:** 2018-09-11

**Authors:** Jennifer L. Dale, Kenneth B. Beckman, Julia L. E. Willett, Jennifer L. Nilson, Nagendra P. Palani, Joshua A. Baller, Adam Hauge, Daryl M. Gohl, Raymond Erickson, Dawn A. Manias, Michael J. Sadowsky, Gary M. Dunny

**Affiliations:** a Department of Microbiology and Immunology, University of Minnesota Medical School, Minneapolis, Minnesota, USA; b University of Minnesota Genomics Center, University of Minnesota, Minneapolis, Minnesota, USA; c Minnesota Supercomputing Institute, University of Minnesota, Minneapolis, Minnesota, USA; d BioTechnology Institute, Department of Soil, Water, and Climate and Department of Plant and Microbial Biology, University of Minnesota, St. Paul, Minnesota, USA; University of Illinois at Chicago

**Keywords:** TnSeq, functional genomics, gut fitness, opportunistic pathogen

## Abstract

Enterococcus faecalis is a common commensal bacterium in animal gastrointestinal (GI) tracts and a leading cause of opportunistic infections of humans in the modern health care setting. E. faecalis OG1RF is a plasmid-free strain that contains few mobile elements yet retains the robust survival characteristics, intrinsic antibiotic resistance, and virulence traits characteristic of most E. faecalis genotypes. To facilitate interrogation of the core enterococcal genetic determinants for competitive fitness in the GI tract, biofilm formation, intrinsic antimicrobial resistance, and survival in the environment, we generated an arrayed, sequence-defined set of chromosomal transposon insertions in OG1RF. We used an orthogonal pooling strategy in conjunction with Illumina sequencing to identify a set of mutants with unique, single *Himar*-based transposon insertions. The mutants contained insertions in 1,926 of 2,651 (72.6%) annotated open reading frames and in the majority of hypothetical protein-encoding genes and intergenic regions greater than 100 bp in length, which could encode small RNAs. As proof of principle of the usefulness of this arrayed transposon library, we created a minimal input pool containing 6,829 mutants chosen for maximal genomic coverage and used an approach that we term SMarT (sequence-defined *mariner* technology) transposon sequencing (TnSeq) to identify numerous genetic determinants of bile resistance in E. faecalis OG1RF. These included several genes previously associated with bile acid resistance as well as new loci. Our arrayed library allows functional screening of a large percentage of the genome with a relatively small number of mutants, reducing potential effects of bottlenecking, and enables immediate recovery of mutants following competitions.

**IMPORTANCE** The robust ability of Enterococcus faecalis to survive outside the host and to spread via oral-fecal transmission and its high degree of intrinsic and acquired antimicrobial resistance all complicate the treatment of hospital-acquired enterococcal infections. The conserved E. faecalis core genome serves as an important genetic scaffold for evolution of this bacterium in the modern health care setting and also provides interesting vaccine and drug targets. We used an innovative pooling/sequencing strategy to map a large collection of arrayed transposon insertions in E. faecalis OG1RF and generated an arrayed library of defined mutants covering approximately 70% of the OG1RF genome. Then, we performed high-throughput transposon sequencing experiments using this library to determine core genomic determinants of bile resistance in OG1RF. This collection is a valuable resource for comprehensive, functional enterococcal genomics using both traditional and high-throughput approaches and enables immediate recovery of mutants of interest.

## Abstract (precis)

The robust ability of Enterococcus faecalis to survive outside the host and to spread via oral-fecal transmission and its high degree of intrinsic and acquired antimicrobial resistance all complicate the treatment of hospital-acquired enterococcal infections. The conserved E. faecalis core genome serves as an important genetic scaffold for evolution of this bacterium in the modern health care setting and also provides interesting vaccine and drug targets. We used an innovative pooling/sequencing strategy to map a large collection of arrayed transposon insertions in E. faecalis OG1RF and generated an arrayed library of defined mutants covering approximately 70% of the OG1RF genome. Then, we performed high-throughput transposon sequencing experiments using this library to determine core genomic determinants of bile resistance in OG1RF. This collection is a valuable resource for comprehensive, functional enterococcal genomics using both traditional and high-throughput approaches and enables immediate recovery of mutants of interest.

## INTRODUCTION

Enterococcus faecalis is normally a gastrointestinal (GI) tract commensal comprising a relatively minor component of the fecal microbiota from healthy adult humans. However, under conditions of GI dysbiosis, especially in immunocompromised patients receiving antibiotic therapy, E. faecalis and related species, such as Enterococcus faecium, can reach abnormally high population densities in the GI tract and cause systemic infections upon escape from their normal intestinal niche (1, 2). It is well known that opportunistic enterococcal infections in health care settings and epidemic spread of certain clones within hospitals correlate with inherent and acquired resistance to antimicrobials as well as the ability of these organisms to survive harsh conditions outside the patient. These traits enhance the persistence of enterococci in the hospital environment and patient-to-patient transmission by fecal-oral routes (3). Despite the pathogenic potential of enterococci in susceptible patients, our understanding of normal enterococcal ecology and the genetic basis for persistence in the GI tract of healthy individuals is limited. It is therefore of great importance to understand the mechanisms by which enterococcal populations interact with the intestinal communities of healthy individuals and how the enterococci exploit dysbiosis to overgrow in the GI tract and escape the intestine to cause systemic infections.

Genomic analysis of commensal and infection-associated E. faecalis strains reveals a highly conserved (in terms of both synteny and protein sequences) core of approximately 2,500 genes, with a preponderance of additional mobile elements (plasmids, transposons [Tns], and genomic islands) encoding antibiotic resistance and suspected virulence determinants involved in hospital outbreaks (4, 5). Recently, an elegant phylogenetic analysis of genome sequences from 24 diverse enterococcal species and five closely related nonenterococcal species suggested that enterococci have coevolved as GI tract commensals with terrestrial animals since the time of their divergence from fish (6). In that report, Lebreton et al. argue that strong evolutionary selection for both colonization and persistence in the GI tract, as well as for resistance to harsh terrestrial environments outside host animals, served to endow enterococci with traits that contribute to their prevalence as opportunistic hospital pathogens. In the case of humans, numerous other mammals, and other invertebrates, including insects, E. faecalis is often the predominant enterococcal GI tract symbiont (3).

Many studies of enterococcal virulence and pathogenesis have understandably focused on gene products encoded by mobile elements identified in clinical isolates from infections (7, 8). In contrast, we and others have identified loci within the conserved core genome that are also required for virulence (9–14). The rationale for such studies is that even “unadorned” strains lacking most mobile elements have an innate ability to colonize the GI tract, have a high degree of intrinsic antibiotic resistance, can form biofilms on host tissues and abiotic surfaces, and can exhibit virulence in experimental infection models designed to replicate opportunistic infections in humans. The core genome thus serves as a critical genetic foundation for continued gene acquisition and evolution of increasingly virulent and antibiotic-resistant strains. Since they are found in virtually all isolates, conserved determinants of colonization, virulence, and antimicrobial resistance in the E. faecalis core genome may be attractive for development of vaccines or anti-infective drugs. E. faecalis strain OG1RF lacks plasmids and many known lysogenic phages and pathogenicity islands found in clinical isolates and thus serves as a model for analysis of the core genome (4). OG1RF carries several conserved gene products contributing to adherence to host tissues, biofilm formation, virulence in experimental infections, and intrinsic resistance to antibiotics. These gene products also include several antigenic determinants that are reactive with convalescent-phase serum from patients with enterococcal bloodstream infections, indicative of their expression in the infected host (15).

The generation of random insertion mutations in bacterial genomes using transposons (Tns) and the subsequent use of these mutant libraries in genetic screens have been an essential tool for functional genomic analysis for over 30 years (16, 17). Our laboratory generated an arrayed collection of approximately 15,000 clones carrying random insertions of a *mariner*-derived transposon in the chromosome of strain OG1RF (18). Amplification and sequencing of Tn-chromosome junctions of randomly chosen clones from this collection suggested that the majority contained single Tn insertions distributed throughout the chromosome. This was further supported by analysis of individual mutants identified in genetic screens for determinants of *in vitro* biofilm formation, virulence in experimental infection models, and intrinsic antibiotic resistance (19–21). However, most transposon insertions in this library have not been mapped, nor are they associated with a specific phenotype.

Recently, numerous studies have documented the power of combining transposon mutagenesis and high-density sequencing (TnSeq) in competition experiments with large pools of random Tn mutants for comprehensive functional genomic analysis (22). Comparison of the relative abundance of mutants in input pools with that in output pools allows for estimation of the contribution of individual genes to bacterial fitness under a given experimental condition; clones carrying mutations in genes important for fitness become selectively less abundant in output pools. An attractive feature of TnSeq is the ability to interrogate the entire genome in a single screen in the context of complex microbial communities. A complicating issue with the use of TnSeq, however, is stochastic loss of mutants under the growth conditions used in the screen. In TnSeq experiments involving plant and animal hosts, it is widely recognized that bottlenecking may be especially problematic in cases where the niche colonized by microbes has a small carrying capacity (22–25). Less recognized is the potential for bottlenecking during the preparation and amplification of libraries of Tn-host junction fragments (26, 27). Therefore, it is of interest to build transposon libraries that cover maximal genomic space with a minimum number of mutants to decrease issues with biological and technical bottlenecking.

Here, we describe the use of a pooling strategy to determine Tn insertion sites in the entire collection of arrayed OG1RF transposon mutants (*n* ≈ 15,000) generated by Kristich et al. (18). We mapped the insertion sites of 10,281 Tn mutants to 8,607 unique TA dinucleotides (the insertion site for *mariner* elements) in the genome. From this set, we assembled a collection of 6,829 clones containing single, unique insertions in more than 70% of the predicted protein-encoding open reading frames in OG1RF and in over 500 intergenic regions >100 bp in length, which could encode small RNA (sRNA) regulators. To facilitate downstream applications, this library is arrayed in 96-well plates, which enables rapid recovery of mutants of interest. As a demonstration of the utility of this collection, we used the arrayed Tn library for a high-throughput sequencing approach that we termed SMarT (sequence-defined *mariner* technology) TnSeq to identify core genomic determinants of bile salt resistance in E. faecalis OG1RF. Our arrayed library can expedite high-throughput functional genetic studies of enterococci by facilitating TnSeq experiments that use small input pools while retaining a high degree of genomic coverage.

## RESULTS

### Orthogonal pooling, sequencing, and Straight Three mapping of transposon insertions in OG1RF.

**FIG 1:**
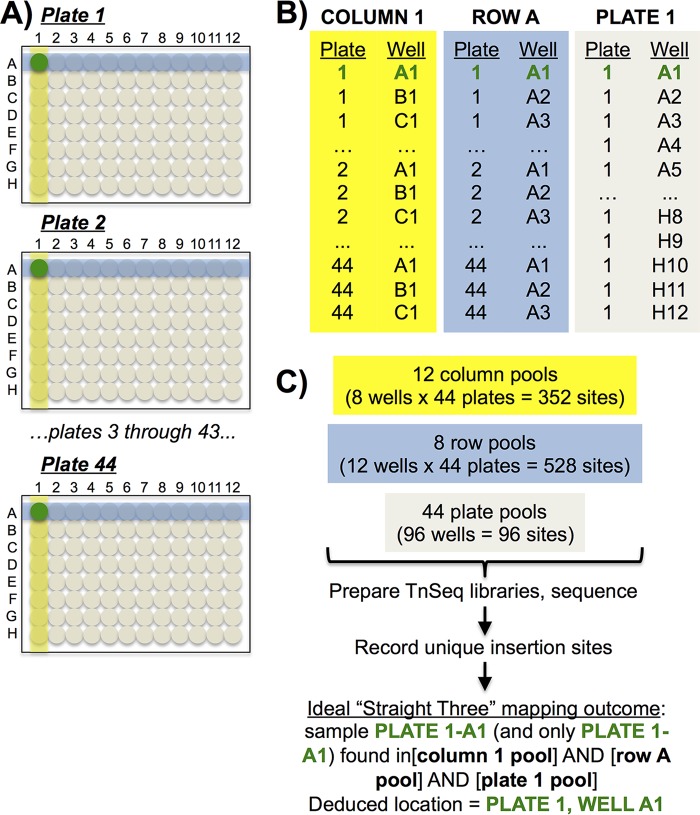
The Straight Three mapping approach to large-scale Tn site mapping in arrayed libraries. The Straight Three algorithm relies on 3D (row × column × plate) orthogonal pooling to map Tn insertion sites. Straight Three mapping is illustrated using the mutant in plate 1, well A1. (A) Pools of mutants were made from columns (yellow shading), rows (blue shading), and full plates. (B and C) Unambiguous assignment of a Tn insertion location to a specific well in a given plate occurs when that position is identified once in a column pool, once in a row pool, and once in a single-plate pool (see, for example, plate 1, well A1). Mutants occupying analogous wells in different plates (for example, plate 2, well A1, and plate 3, well A1) are sorted based on their presence in the plate pools. For examples of results obtained when multiple clones containing Tn insertions at the same site are present in a single plate or when multiple Tn insertions are present in the same well, see Fig. S1.

**FIG 2:**
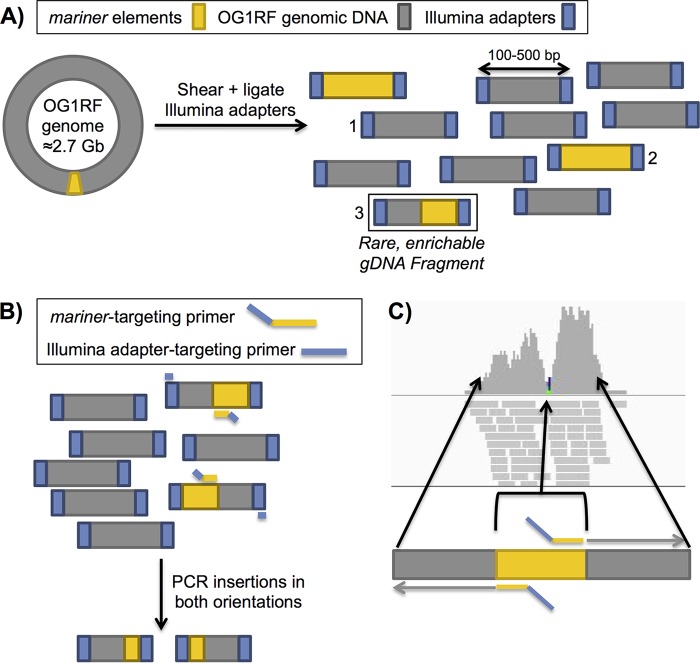
Library preparation and mapping of Tn insertion sites. (A) Next-generation sequencing libraries prepared from OG1RF *mariner* Tn mutant DNA potentially contain three groups of genomic DNA fragments: 1, genomic DNA without Tn sequence (gray); 2, Tn-only sequence (yellow); and 3, genomic DNA-Tn junctions (gray and yellow). Only genomic DNA (gDNA)-Tn junctions (≈0.01% of library fragments) will be effectively enriched via TnSeq. (B) TnSeq amplification using primers that recognize Illumina library adapters (blue) and the 3′ ends of *mariner* elements selectively amplifies regions that contain genomic DNA-Tn junctions. Sequences on either side of the Tn insertion are identified due to bidirectional amplification of the *mariner* element. (C) Mapping of reads obtained from Illumina sequencing to the OG1RF genome identifies the precise TA site of a Tn insertion.

To facilitate rapid and efficient determination of the insertion sites in the entire collection of Tn mutants (*n* ≈ 15,000) generated by Kristich et al. (18), we developed the heuristic Straight Three strategy for orthogonal pooling and mapping (Fig. 1). This allows for precise definition of the insertion sites of a large number of arrayed Tn insertions using a minimal number of sequencing reactions. The Tn mutants were arrayed in 177 plates, each containing 96 storage tubes with unique 10-digit barcodes that can be scanned by robotic tube handlers. The collection of plates was divided into four pools of ∼44 racks each. Pools were generated from sets of common rows (A to H) and columns (1 to 12) from all 44 plates in a set, and all mutants from a single plate were combined to create plate pools (44 plates per set). Genomic DNA was extracted from each pool, and fragments containing a chromosome-Tn junction were enriched (Fig. 2A and B), sequenced, and mapped to the OG1RF genome (Fig. 2C).

10.1128/mSystems.00062-18.4 FIG S1 Troubleshooting scenarios for Straight Three Tn insertion site mapping. (A) Scenario 1: sibling clones (clones with identical Tn insertion sites, indicated by green dots) occur in the same plate. As shown in the graph, an equivalent number of reads (*n*) mapping to the Tn mutant in well A1 will be present in the row A and column 1 pools. Approximately 2*n* reads mapping to the Tn mutant in well A1 will be present in the plate 1 pool, as two insertions at this site are present in the plate. (B) Scenario 2: some wells contain Tn insertions that map to >1 unique TA site in the genome (indicated by maroon and coral coloring in well A1). Then, Straight Three mapping will identify reads associated with 2 TA sites (mock sequences shown, TA insertion sites shown in maroon and coral) in well A1. If this duplicity is due to the presence of >1 distinct strain, each mutant can be isolated from single colonies grown on agar plates. Download FIG S1, PDF file, 0.4 MB. Copyright © 2018 Dale et al. 2018 Dale et al. This content is distributed under the terms of the Creative Commons Attribution 4.0 International license.

Unambiguous Straight Three mapping occurs when reads corresponding to a single Tn insertion in a single plate well with no sibling clones in the library appear in relatively equal abundance in exactly 3 pools (one column, one row, and one plate). An example of this is illustrated in Fig. 1A. Although mutants in position A1 of other plates in this set also appear in the column 1 and row A pools (Fig. 1A and B), they do not appear in the plate 1 pool. Similarly, other mutants in row A (Fig. 1A, blue boxes) or column 1 (Fig. 1A, yellow boxes) appear in two of the pools but not all three. Additional information regarding data refinement and mapping scenarios can be found in Materials and Methods as well as Fig. S1 and Text S1 in the supplemental material.

10.1128/mSystems.00062-18.3 TEXT S1 The Straight Three approach to identifying arrayed Tn mutants. Description of the mapping and filtering steps used to map the arrayed Tn mutants. Download Text S1, PDF file, 0.7 MB. Copyright © 2018 Dale et al. 2018 Dale et al. This content is distributed under the terms of the Creative Commons Attribution 4.0 International license.

### Transposon insertion coverage of the OG1RF genome.

**FIG 3:**
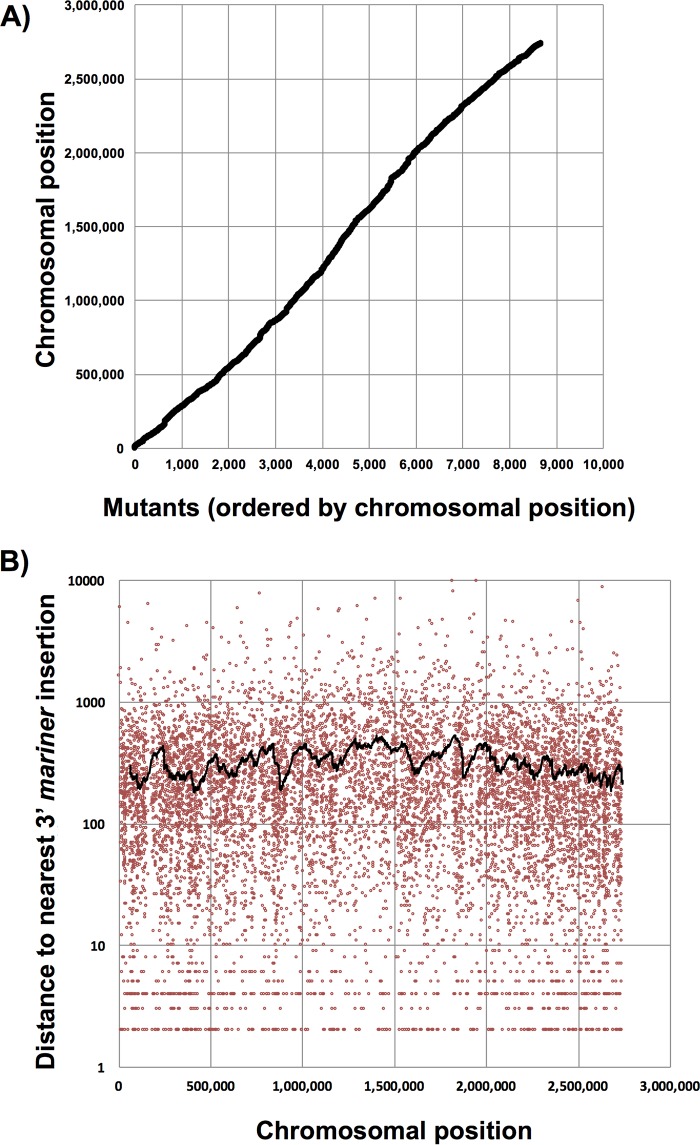
Distribution of mapped Tn insertions in the E. faecalis OG1RF genome. (A) Linear distribution of all mapped Tn mutants (black dots) ordered by position in the chromosome. (B) The distance between Tn mutants (red dots) was plotted relative to chromosomal position. The interinsertional spacing between transposon mutants is relatively constant across the OG1RF genome (316 ± 580 bp). The black line represents the running average of Tn spacing (with a 50-bp sliding window) for any given chromosomal position.

Using the Straight Three approach, we mapped 10,281 Tn mutants from the original collection of ≈15,000 clones and identified insertions at 8,607 unique TA positions in the OG1RF genome. The majority of mapped plate wells (8,016 of 9,139) contained a single Tn insertion mutant. Reads mapping to two positions were identified in 1,834 wells, and reads mapping to three or more positions were found in 431 wells. A complete summary of mapped transposon insertions is provided in Table S1. Insertions were linearly distributed on a genome-wide scale (Fig. 3A) and had an average interinsertional distance of 316 ± 580 bp (Fig. 3B). Tn insertions were detected in approximately 72% of the predicted protein-encoding open reading frames in OG1RF (1,912 of 2,658) and in over 500 intergenic regions >100 bp in length (568 of 997). Additionally, we identified Tn insertions in 390 of the 547 hypothetical genes (determined from *old_locus_tag* identifiers in NCBI reference genome NC_017316.1).

10.1128/mSystems.00062-18.7 TABLE S1 Mapped OG1RF Tn mutants. Information for the complete set of E. faecalis OG1RF Tn mutants mapped by the Straight Three approach. Download Table S1, XLSX file, 0.6 MB. Copyright © 2018 Dale et al. 2018 Dale et al. This content is distributed under the terms of the Creative Commons Attribution 4.0 International license.

**FIG 4:**
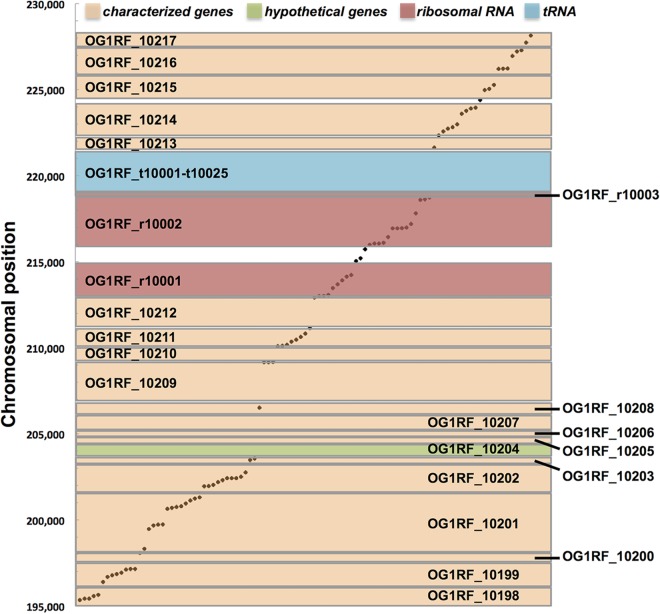
Distribution of Tn insertions across a subset of genes. Tn insertions (dots) mapped to the genomic region from OG1RF_10198 (*aldA*, aldehyde dehydrogenase) through OG1RF_10217 (phosphoglycerate mutase). The *y* axis indicates the chromosome position. Each rectangle spanning the *x* axis represents a different genomic feature (characterized protein-encoding genes [orange], hypothetical genes [green], intergenic regions [white], rRNA [red], and tRNA [teal]). In loci containing insertions, Tn elements are evenly distributed (distribution throughout the length of each feature is represented by the position on the *y* axis). For simplicity, the intergenic identifiers are not shown.

To illustrate the distribution of Tn insertions in single genes, a region of the chromosome spanning genes OG1RF_10198 to OG1RF_10217 was examined in detail (Fig. 4). This region contains a variety of genomic features, including known genes, hypothetical genes, intergenic regions, one of four rRNA operons in OG1RF, and 25 of the 58 tRNAs carried by E. faecalis. We asked whether Tn insertions were evenly dispersed throughout these features or whether our Tn library generation method resulted in regions of enriched integrations. We observed Tn insertions in all classes of genomic features except for the tRNA operon and the hypothetical gene OG1RF_10204. In genes that contained insertions, Tn elements were typically distributed throughout the coding region in a linear manner. Several characterized protein-encoding regions lacked Tn insertions, including OG1RF_10205 (cell division protein DivIC), OG1RF_10209 (ATP-dependent metalloprotease FtsH), and OG1RF_10212 (lysyl-tRNA synthetase). Although our current study lacks the statistical power necessary to predict essential genes, these gene products have been previously shown to be essential for cell growth or division in a variety of microbes (28).

### SMarT TnSeq identification of core genomic determinants of bile resistance.

Using our sequenced collection of 10,281 OG1RF transposon mutants, we constructed a smaller library consisting of 6,829 unique mutants (Table S2) arrayed in 96-well microtiter plates with the goal of creating a defined library that achieves high levels of genomic coverage with a reduced number of mutants. The library contains at least one Tn insertion in approximately 68% of OG1RF open reading frames (1,809 of 2,658) and in 620 intergenic regions. Mutants were chosen for inclusion in the SMarT TnSeq library based on Tn position in the gene, unique insertion sites, and high-confidence mapping results. Mutants were excluded if multiple Tn insertions were detected in a single well of the arrayed library. The 6,829 strains were reracked from the entire arrayed population of OG1RF mutants using a robotic tube handler, cultured individually on solid growth medium, and combined manually to create a SMarT TnSeq input pool with maximal genomic coverage from a small set of mutants for fitness screens.

10.1128/mSystems.00062-18.8 TABLE S2 SMarT TnSeq library. Information for all mutants chosen from the complete arrayed library for inclusion in the E. faecalis OG1RF SMarT TnSeq library. Download Table S2, XLSX file, 0.5 MB. Copyright © 2018 Dale et al. 2018 Dale et al. This content is distributed under the terms of the Creative Commons Attribution 4.0 International license.

We used the SMaRT TnSeq library to gain insight into a trait that is essential for E. faecalis survival in a host. To colonize and thrive in the GI tract, E. faecalis must withstand harsh physiochemical conditions, including the presence of bile salts. Although previous studies have described enterococcal transcriptional and proteomic responses to bile stress (29–36), no comprehensive, transposon-based experiments to examine the core genomic determinants of bile resistance have been described in OG1RF. Cholic acid, a primary bile acid, is conjugated to taurine or glycine in the liver and secreted into the intestine (37), where it is converted to the secondary bile acid deoxycholic acid by selective GI tract microbiota (38–40). Changes in the level of cholic acid present in the GI tract are associated with restructuring of the gut microbiota (41, 42). Therefore, we chose to study the genes required for resistance to cholic acid using SMarT TnSeq.

**FIG 5:**
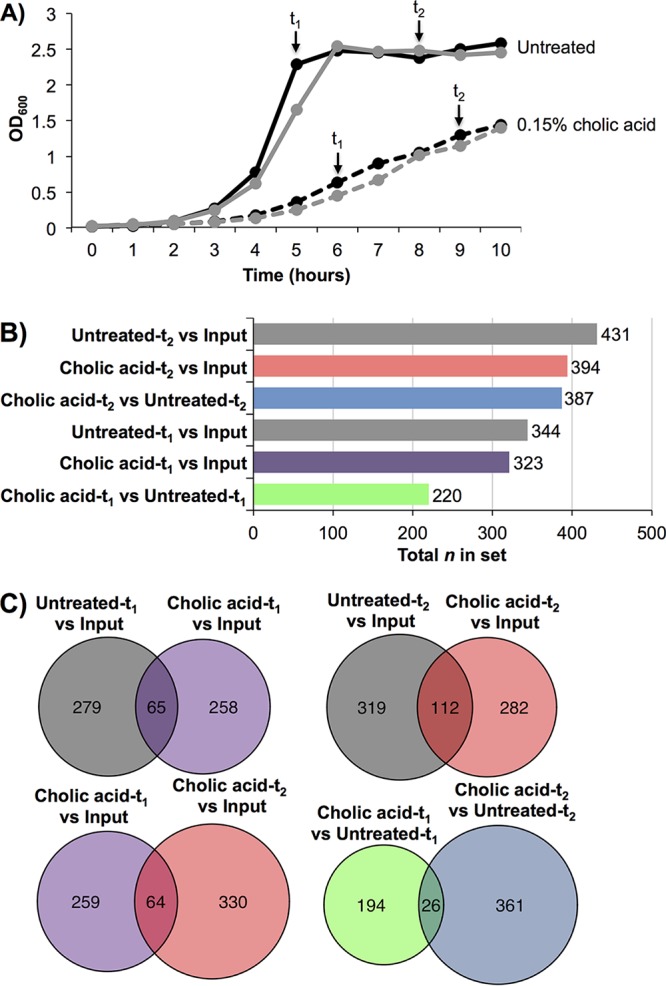
SMarT TnSeq analysis of OG1RF growth in cholic acid. (A) Growth of the SMarT TnSeq library. Samples were taken from untreated cultures (solid lines) or cultures treated with 0.15% cholic acid (dashed lines). Cells were harvested at early and late time points (*t*_1_ and *t*_2_, respectively). Sampling points are shown with arrows. Black and gray lines indicate separate biological replicates. (B) The total number of TA sites significantly underrepresented in each comparison (*P* < 0.05, log_2_ fold change < 0). The cholic acid-specific pairwise comparisons are shown as different colors. (C) TA sites shared between comparisons. Cholic acid-specific comparisons are colored as in panel B. Comparisons between untreated cells and the input sample were included to evaluate the number of mutants with general growth defects.

The growth of OG1RF in liquid culture was reduced but not completely inhibited by 0.15% cholic acid (Fig. S2). We inoculated MM9-YEG (described in Materials and Methods) supplemented with 0.15% cholic acid (or MM9-YEG alone) with the SMaRT TnSeq library and harvested cells from the input pool as well as early and late time points (*t*_1_ and *t*_2_, respectively) (Fig. 5A, sampling points indicated by black arrows). These roughly correspond to late logarithmic and stationary phases of the parental OG1RF strain, respectively. Total DNA was extracted and prepared for Illumina sequencing, and the Tn junctions were mapped to the OG1RF genome. We determined the relative abundance of all mutants in the SMarT TnSeq library under each culture condition and identified mutations that were significantly underrepresented in the presence of cholic acid (adjusted *P* value < 0.05, log_2_ fold change < 0) (Fig. 5B and Table S3). Compared to their relative abundance in the SMarT TnSeq input pool, 323 Tn mutants were significantly underrepresented in the presence of cholic acid at *t*_1_ and 394 Tn mutants were underrepresented in the presence of cholic acid at *t*_2_. Compared to the untreated cultures, 220 Tn mutants from the cholic acid-treated sample were underrepresented at *t*_1_ and 387 Tn mutants were underrepresented at *t*_2_. There was some overlap in mutants with defects in cholic acid; 64 Tn mutants were underrepresented at both time points relative to the input pool, and 26 were underrepresented at both time points relative to the untreated samples (Fig. 5C).

10.1128/mSystems.00062-18.9 TABLE S3 Cholic acid SMarT TnSeq. Output data for all samples from the cholic acid SMarT TnSeq experiments. Download Table S3, XLSX file, 2.7 MB. Copyright © 2018 Dale et al. 2018 Dale et al. This content is distributed under the terms of the Creative Commons Attribution 4.0 International license.

10.1128/mSystems.00062-18.5 FIG S2 Growth of E. faecalis OG1RF in cholic acid. Overnight cultures of OG1RF were adjusted to an OD_600_ of 0.05 in MM9-YEG with or without 0.15% cholic acid. Growth was tracked by measuring the OD_600_ of an aliquot every hour. Data shown are the average from two biological replicates. Download FIG S2, PDF file, 0.03 MB. Copyright © 2018 Dale et al. 2018 Dale et al. This content is distributed under the terms of the Creative Commons Attribution 4.0 International license.

To determine how many of these mutants had general (and not bile-specific) growth defects, we identified insertions that were underrepresented in untreated cultures compared to the input pool. Three hundred forty-four Tn mutants were significantly underrepresented in the untreated sample at *t*_1_ compared to 431 Tn mutants in the untreated sample at *t*_2_ (Fig. 5B). Relative to the input pool, 65 Tn insertions were underrepresented in cholic acid-treated and untreated *t*_1_ samples, and 112 Tn insertions were underrepresented in both *t*_2_ samples (Fig. 5C). A majority of the Tn mutants that were significantly underrepresented in the presence of cholic acid did not have general growth defects, indicating that their phenotypes are due to the presence of cholic acid.

We used a log_2_ fold change cutoff of −2 to identify Tn mutants with the most severe growth defects in the presence of cholic acid (*n* = 26) (Table 1). These included insertions in genes involved in DNA repair (*radA*) and stress response (*hrcA*, *grpE*, *dnaK*, and *dnaJ*), which is in agreement with previously published work demonstrating that bile salts inhibit cell growth by damaging cell walls and DNA (38, 43). DnaK and DnaJ are chaperones that modulate cellular responses to stress such as heat, antibiotics, and ethanol (31, 44), and DnaK was predicted by expression analysis to have a role in E. faecalis bile resistance (33, 34). Additionally, we found that Tn insertions in *aad* (aldehyde/alcohol dehydrogenase), *czrA* (metal transport repressor), and *gcdB* (glutaconyl coenzyme A [CoA] decarboxylase) were significantly underrepresented in the presence of cholic acid; these genes were differentially regulated in the presence of bile in other studies (33–35). These results demonstrate that the SMarT TnSeq library can be used to identify known bile-sensitive mutants as well as subtle cholic acid-specific growth defects that have not been described previously.

**TABLE 1:** Tn mutants identified by SMarT TnSeq as significantly underrepresented in the presence of cholic acid^a^

Position	Locus tag	Gene	Gene product	Log_2_ fold change (adjusted *P* value)			
				Cholic acid (*t*_1_)/input	Cholic acid (*t*_1_)/untreated (*t*_1_)	Cholic acid (*t*_2_)/input	Cholic acid (*t*_2_)/untreated (*t*_2_)
45155	OG1RF_10039	*radA*	DNA repair protein RadA	−0.71 (1.89E−39)	−0.87 (2.81E−24)	**−2.12 (6.68E−141)**	−2.17 (NS)
45325	OG1RF_10039	*radA*	DNA repair protein RadA	−0.64 (1.82E−41)	−1.12 (1.55E−21)	**−2.33 (1.05E−158)**	**−2.54 (6.45E−8)**
266158	OG1RF_10257	*ezrA*	Septation ring formation regulator EzrA	−0.80 (1.02E−42)	−0.64 (1.17E−22)	**−2.34 (8.33E−108)**	−2.30 (NS)
665366	OG1RF_10627	*aad*	Aldehyde-alcohol dehydrogenase	−1.47 (9.81E−6)	−0.92 (9.18E−13)	**−2.29 (1.19E−54)**	−1.61 (5.93E−7)
665450	OG1RF_10627	*aad*	Aldehyde-alcohol dehydrogenase	−1.61 (NS)	−0.48 (0.033)	**−2.18 (5.39E−11)**	−1.17 (6.64E−28)
1118301	Intergenic_1127			−2.06 (NS)	−1.34 (NS)	**−2.29 (1.45E−10)**	−0.81 (0.0026)
1118585	OG1RF_11076	*hrcA*	Heat-inducible transcription repressor HrcA	−2.77 (NS)	−1.84 (5.38E−37)	**−2.72 (3.92E−23)**	**−2.01 (5.29E−15)**
1118707	OG1RF_11076	*hrcA*	Heat-inducible transcription repressor HrcA	−2.76 (NS)	−1.43 (8.98E−16)	**−3.47 (1.77E−13)**	**−2.48 (1.6E−21)**
1119789	OG1RF_11077	*grpE*	Cochaperone GrpE	−2.08 (NS)	−0.59 (1.82E−11)	**−3.22 (4.01E−9)**	**−2.50 (1.09E−22)**
1120304	OG1RF_11078	*dnaK*	Chaperone DnaK	−1.83 (NS)	−1.44 (4.07E−17)	**−2.40 (8.07E−19)**	−2.21 (NS)
1121988	Intergenic_1130			−1.89 (NS)	−1.24 (1.31E−16)	**−2.76 (8.1E−33)**	−2.16 (2.04E−5)
1122948	OG1RF_11080	*dnaJ*	Chaperone DnaJ	−2.11 (NS)	−1.60 (5.16E−10)	**−2.42 (5.01E−22)**	−1.67 (NS)
1213789	OG1RF_11160	NA	Thioesterase	−1.12 (1.9E−21)	0.60 (NS)	**−2.98 (3.05E−5)**	−1.10 (1.16E−48)
1496094	OG1RF_11434	*lspA*	Signal peptidase II	−1.33 (2.05E−5)	−0.36 (NS)	**−3.06 (5.66E−12)**	−1.89 (0.025)
1497213	Intergenic_1490			−0.90 (1.74E−8)	−0.33 (2.5E−4)	**−2.02 (8.52E−19)**	−1.79 (0.0034)
1707088	OG1RF_11636	*relA*	GTP diphosphokinase	−0.85 (8.27E−4)	−0.094 (NS)	**−2.76 (3.37E−4)**	−2.37 (NS)
1793746	OG1RF_11714	NA	Group 2 glycosyltransferase	−0.82 (6.87E−9)	−0.059 (NS)	**−2.31 (7.44E−10)**	−1.62 (0.0051)
1955393	OG1RF_11854	*czrA*	Metal transport repressor protein CzrA	−0.96 (6.74E−8)	−0.44 (NS)	**−2.11 (4.8E−22)**	−1.54 (NS)
1955400	OG1RF_11854	*czrA*	Metal transport repressor protein CzrA	−1.23 (4.48E−6)	−0.40 (NS)	**−2.36 (2.89E−16)**	−1.80 (1.11E−9)
2099505	OG1RF_11987	*atpG*	ATP synthase F_1_ sector gamma subunit	**−2.13 (4.14E−6)**	−0.85 (2.48E−11)	**−3.27 (5.25E−37)**	**−2.08 (8.67E−45)**
2274738	OG1RF_12155	NA	Brp/Blh family beta- carotene 15,15- monooxygenase	−1.08 (1.00E−4)	−1.27 (5.73E−25)	**−2.05 (1.15E−63)**	−2.15 (NS)
2274991	OG1RF_12155	NA	Brp/Blh family beta- carotene 15,15- monooxygenase	−2.20 (NS)	**−2.17 (1.17E−56)**	**−2.75 (3.91E−93)**	−2.49 (NS)
2275412	OG1RF_12155	NA	Brp/Blh family beta- carotene 15,15- monooxygenase	−1.12 (3.41E−18)	−1.20 (3.68E−26)	**−2.10 (1.88E−145)**	−1.82 (NS)
2616803	OG1RF_12481	NA	Choline binding protein	1.10 (NS)	−1.13 (NS)	**−2.87 (1.34E−8)**	−3.60 (NS)
2696898	OG1RF_12538	*guaB*	IMP dehydrogenase	−1.58 (NS)	−1.24 (5.24E−5)	**−2.10 (4.36E−27)**	−1.41 (NS)
2731478	OG1RF_12569	*gcdB*	Glutaconyl-CoA decarboxylase	−1.21 (6.05E−5)	−0.49 (NS)	**−2.90 (8.84E−14)**	−1.98 (NS)

a The log_2_ fold changes are in relative abundance between the two indicated conditions. For adjusted *P* values, an adjusted *P*  of < 0.05 is significant; NS indicates “not significant.” Boldface values indicate the most extremely underrepresented mutants (log_2_ fold changes less than −2). The position column indicates the first nucleotide position of the Tn insertion site. NA, not assigned.

To gain insight into the usefulness of including multiple Tn insertions within the same gene in SMarT TnSeq libraries, we examined the Tn mutants with the most severe bile-associated defects (Table 1 and Table S3). Four genes contained multiple Tn insertions with severe defects (log_2_ fold change less than −2) under at least one bile-specific condition (*radA*, *aad*, *hrcA*, *czrA*, and OG1RF_12155). Additional genes had multiple Tn insertions with defects in the presence of cholic acid, but the magnitude of the defect was not as severe (−2 < log_2_ fold change < 0). However, other genes with significant cholic acid-associated defects had additional Tn insertions that did not share the same phenotype. For example, the Tn insertion at genomic position 1707088 in *relA* is underrepresented in the cholic acid-treated samples relative to the input pool (Table 1). The SMarT TnSeq library contains an additional insertion in *relA* at genomic position 1704953. The abundance of this Tn mutant was not significantly different in the presence of cholic acid relative to the input pool, and it was slightly overrepresented relative to untreated samples (Table S3). Similarly, the arrayed library contains multiple Tn insertions in OG1RF_12481, and the abundance of most of these did not significantly change in the presence of cholic acid. Mutants without a phenotype may produce functional protein fragments. Therefore, while some genes with multiple independent Tn insertions have shared phenotypes across all mutants, inclusion of *n* > 1 Tn mutants in a given locus may be informative about gene structure and function.

**FIG 6:**
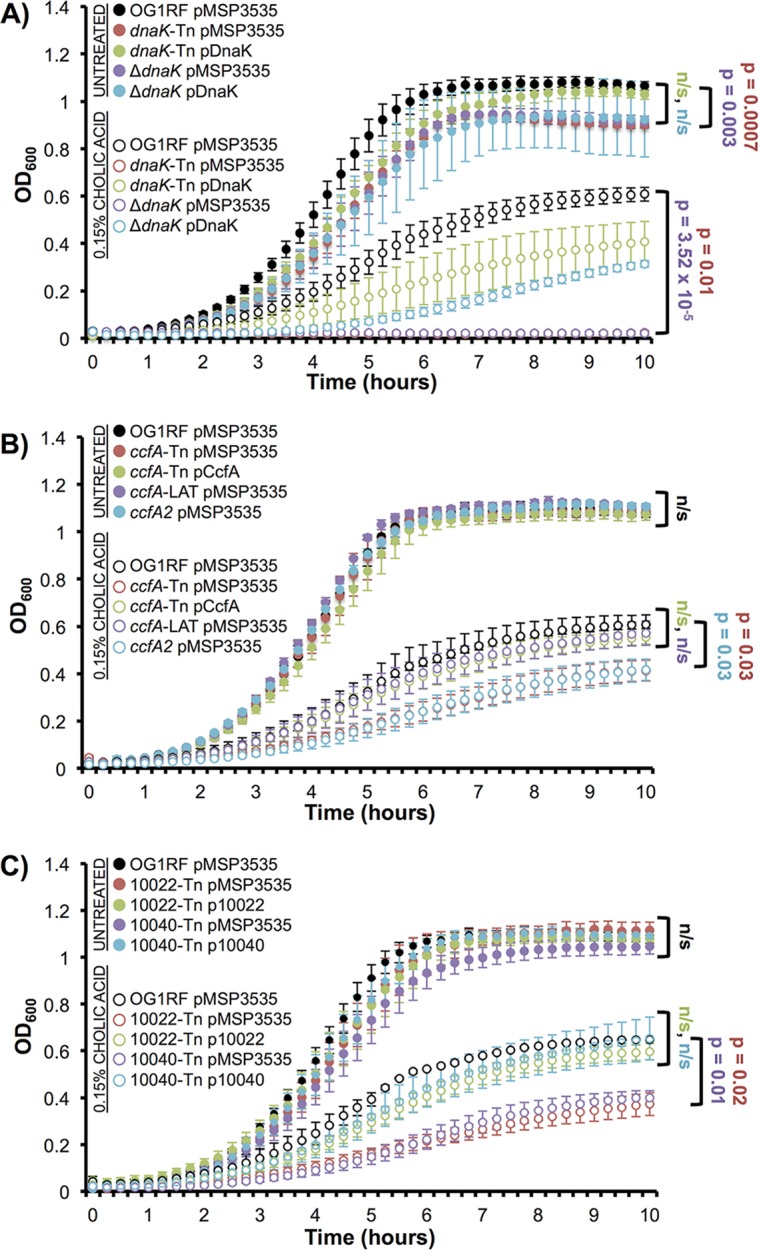
Cholic acid sensitivity of Tn mutants identified by SMarT TnSeq. All strains were grown in MM9-YEG–0.15% cholic acid (or MM9-YEG medium alone for untreated samples). (A) Strains with *dnaK* mutations (transposon insertion, *dnaK*-Tn; markerless deletion, Δ*dnaK*) do not grow in the presence of cholic acid (open purple and red circles). Expression of wild-type *dnaK* from a plasmid (*dnaK*-Tn pDnaK, open green circles; Δ*dnaK* pDnaK, open teal circles) significantly rescues growth relative to the vector control strains. (B) Mutations that disrupt the translation of CcfA (Tn insertion, *ccfA*-Tn; stop codon, *ccfA*2) significantly reduce survival in cholic acid. Altering the cCF10 pheromone sequence to an inactive variant via an alanine substitution (*ccfA*-LAT) does not alter survival in cholic acid compared to OG1RF (compare open purple and black circles). In the untreated condition, none of the mutant strains had significant differences in growth relative to OG1RF (filled circles). (C) Strains with Tn insertions in hypothetical proteins (10022-Tn and 10040-Tn) have significantly reduced survival in the presence of cholic acid (open red and purple circles). These growth defects are complemented with a plasmid-borne copy of the genes (open green and teal circles, respectively). Values plotted at each time point are the averages from three biological replicates (each with two technical replicates). Error bars are standard errors of the means. Significant differences between OD_600_ values at the end of the experiment (*t* = 10 h) were determined using a two-tailed Student *t* test (*P* < 0.05).

To validate the cholic acid-associated growth defects detected by SMarT TnSeq, we compared the growth of individual mutants to wild-type OG1RF in monoculture growth experiments (Table 2 and Fig. S3). We selected diverse mutants based on (i) the extent of previous gene characterization (annotated gene product versus hypothetical), (ii) whether their role in bile resistance was previously identified, and (iii) the severity of the phenotype (based on log_2_ fold change) identified using SMarT TnSeq. Mutants were isolated from the arrayed SMarT TnSeq stock plates, and growth was measured via values of optical density at 600 nm (OD_600_). We first examined a transposon mutant with an insertion in *dnaK* (OG1RF_11078). Although the *dnaK* mutant strains had a small growth defect compared to OG1RF in the absence of cholic acid, this was greatly enhanced in the presence of cholic acid (Fig. 6A, compare filled and open circles). The *dnaK* transposon insertion (*dnaK*-Tn) carrying an empty pMSP3535 plasmid vector failed to grow in the presence of 0.15% cholic acid (Fig. 6A, open red circles), and growth was partially restored with expression of *dnaK* from a nisin-inducible plasmid (pDnaK) (Fig. 6A, open green circles). Importantly, this transposon mutant had the same phenotype as the isogenic *dnaK* deletion strain (Fig. 6A, open purple and blue circles), demonstrating that the transposon mutant strains in our SMarT TnSeq library can recapitulate phenotypes associated with markerless gene deletions.

**TABLE 2:** Underrepresented Tn mutants chosen for further analysis^a^

Position	Locus tag	Gene	Gene product	Log_2_ fold change (adjusted *P* value)			
				Cholic acid (*t*_1_)/input	Cholic acid (*t*_1_)/untreated (*t*_1_)	Cholic acid (*t*_2_)/input	Cholic acid (*t*_2_)/untreated (*t*_2_)
26611	OG1RF_10022	NA	Hypothetical protein	−0.49 (NS)	−0.62 (3.48E−5)	−1.32 (NS)	−1.25 (1.01E−30)
46306	OG1RF_10040	NA	PIN-domain protein	−0.88 (5.49E−35)	−1.02 (9.86E−17)	−1.89 (NS)	−2.01 (3.91E−104)
1120304	OG1RF_11078	*dnaK*	Chaperone DnaK	−1.83 (4.07E−17)	−1.44 (NS)	−2.4 (NS)	−2.21 (8.07E−19)
2738340	OG1RF_12576	*ccfA/spoIIIJ*	Stage III sporulation protein J	−1.08 (3.52E−29)	−1.23 (7.56E−4)	−1.82 (NS)	−1.87 (1.32E−76)

a The log_2_ fold changes are in relative abundance between the two indicated conditions. For adjusted *P* values, adjusted *P* < 0.05 is significant; NS indicates not significant. The position column indicates the first nucleotide position of the Tn insertion site. NA, not assigned.

10.1128/mSystems.00062-18.6 FIG S3 Mutants chosen for monoculture cholic acid experiments. The colored shapes represent genes with Tn insertions that had significantly reduced abundance in the presence of cholic acid as evaluated by TnSeq. Adjacent black shapes represent upstream and downstream genes. Filled black triangles indicate the Tn insertion site. Numbers below the genes are OG1RF gene identifiers (based on *old_locus_tag* identifiers from reference genome NC_017316.1). Download FIG S3, PDF file, 0.5 MB. Copyright © 2018 Dale et al. 2018 Dale et al. This content is distributed under the terms of the Creative Commons Attribution 4.0 International license.

SMarT TnSeq analysis revealed that a transposon insertion in *spoIIIJ*/*ccfA* (OG1RF_12576), which encodes a lipoprotein belonging to the YidC/Oxa1 family of insertases, was significantly underrepresented in the presence of cholic acid. CcfA is also the protein from which the peptide pheromone cCF10 is derived; cCF10 modulates a complex regulatory network that controls conjugative transfer of the E. faecalis plasmid pCF10 (45). Therefore, we sought to determine whether the cholic acid growth phenotype of the *ccfA* transposon mutant (*ccfA*-Tn) was related to alteration of cCF10 production or to abolished expression of the mature CcfA protein. In monoculture, the growth of *ccfA*-Tn carrying an empty pMSP3535 vector was reduced in the presence of cholic acid (Fig. 6B, open red circles) relative to the growth of OG1RF (Fig. 6B, open black circles). A plasmid-borne copy of *ccfA* (pCcfA) complemented the growth defect (Fig. 6B, open green circles). Next, we examined the growth of a previously generated strain in which the cCF10 pheromone sequence was altered via a single nucleotide substitution (*ccfA*-LAT, MKKYKRLLMAGL[V→A]TLVFV) (46, 47). The cCF10 variant peptide produced by this strain lacks pheromone activity, but the amino acid sequence of the mature CcfA protein is not disrupted. Relative to OG1RF, the growth of *ccfA*-LAT carrying an empty plasmid was not significantly reduced in the presence of cholic acid (Fig. 6B, open purple circles), indicating that the cholic acid phenotype is independent of cCF10 pheromone production. A *ccfA* mutant strain in which the first two residues of cCF10 were replaced by stop codons (*ccfA*2, MKKYKRLLMAG**) (47) has reduced growth in the presence of cholic acid (Fig. 6B, open blue circles). Like the transposon mutant, this strain makes neither functional cCF10 nor full-length CcfA. None of the *ccfA* mutations significantly alter growth relative to OG1RF in the absence of cholic acid (Fig. 6B, filled circles). CcfA belongs to the YidC/Oxa1 family of insertases, so it may be involved in folding or insertion of membrane proteins required for survival during bile stress. Taken together, these data show that the mature CcfA protein (but not cCF10 peptide) is required for resistance to cholic acid. Importantly, a role in bile resistance has never been ascribed to this protein.

Although many of the SMarT TnSeq transposon insertions that were underrepresented in the presence of cholic acid are found in genes with annotated functions, additional underrepresented mutations lie within hypothetical, uncharacterized genes (Table S3). SMarT TnSeq revealed that transposon insertions in OG1RF_10022 and OG1RF_10040 led to a fitness defect in the presence of bile. OG1RF_10022 is a hypothetical protein containing a domain of unknown function (DUF956). OG1RF_10040 has computationally predicted nucleic acid binding domains, so it may be involved in the expression of genes required for survival in the presence of bile salts or in the mammalian intestinal tract. In the presence of 0.15% cholic acid, the growth of OG1RF_10022-Tn and OG1RF_10040-Tn carrying an empty pMSP3535 plasmid was reduced relative to OG1RF (Fig. 6C, open red and purple circles), and expression of the respective wild-type gene from a plasmid (pMSP3535::OG1RF_10022 or pMSP3535::OG1RF_10040) restored growth (Fig. 6C, open green and teal circles). Taken together, the cumulative results from our SMarT TnSeq analysis demonstrate that both new and previously identified genetic determinants of bile resistance can be discovered using this approach.

## DISCUSSION

### Orthogonal pooling approach for mapping insertions in OG1RF.

The creation of arrayed libraries containing gene deletions or insertional mutations has rapidly advanced the pace at which bacterial genetic experiments can be performed. Early iterations of arrayed Tn resources relied on the identification of single mutants via Sanger sequencing, which is a laborious and costly undertaking (24, 48, 49). Fu et al. (24) reported one of the first examples of a sequence-defined transposon library in their analysis of genetic determinants for intestinal colonization in Vibrio cholerae, utilizing an input library of 3,156 mutants with single insertions in most nonessential V. cholerae genes. Tn mutants were chosen based on individual sequencing results of approximately 23,000 randomly isolated mutants. A three-dimensional (3D) (row/column/plate) pooling approach was also used to identify mutants in arrayed libraries via PCR by combining Tn-specific and gene-specific primers to identify insertions in genes of interest (50).

High-throughput pooling and mapping developments allow for simultaneous identification of a large number of Tn mutants that can be traced to specific stocks, and they are faster and less expensive than sequencing individual mutants at this scale (51–53). Recent examples of these are INSeq (54, 55), Knockout Sudoku (56, 57), and Cartesian pooling-coordinate sequencing (CP-CSeq) (58). INSeq uses a robotic liquid handling system to create mutant pools. Although this approach requires access to specific equipment, it is less susceptible to errors during pooling, and the mapping technique can easily handle the presence of sibling clones in an arrayed library. In contrast, Knockout Sudoku, CP-CSeq, and our Straight Three method use a simpler pooling strategy that can be performed manually or robotically, making them more adaptable by researchers without access to robotic pooling equipment. While manual pooling increases the risk of error, Straight Three mapping implements a quality control mechanism in the form of randomized, known blank wells in each plate. Therefore, mispooling would be simple to detect, as no Tn insertions should map to these blank wells.

In Straight Three mapping, the number of plate pools scales linearly with the addition of library plates. However, this increase is relatively small considering the number of mutants that can be mapped with the addition of one more pool, and the plate pool increase does not impose a significant constraint on the overall sequencing and mapping process. The number of pools needed to sequence a library of approximately 17,000 mutants (∼240 libraries total) is still 2 orders of magnitude less than the number of sequencing reactions (∼17,000) needed to sequence all mutants individually. Other mapping approaches that require generation of a pool from all wells of a single plate also scale in this manner.

Common troubleshooting issues have been identified with other pooling/mapping methods and our Straight Three approach. The first is that some Tn mutants, at first pass, cannot be mapped to a specific library location unambiguously. For Straight Three mapping, this may be due to identification in more than three pools, or it may be caused by variations in relative abundance of sequencing reads between pools. These specific scenarios can typically be resolved with iterative data cleaning. In our study, we used a combination of manual and computational refinement to identify the original locations of mutants that could not be solved by immediate Straight Three mapping. Knockout Sudoku utilizes a Bayesian interference algorithm to determine sibling Tn mutant locations computationally.

Of the 9,139 individual wells to which we were able to map Tn mutants, approximately 1,400 of them contained 2 or more Tn insertions. We have observed anecdotally that using robotic colony pickers can be a major source of this contamination. Therefore, we consider the “best practice” of arrayed Tn library construction to be manual distribution of Tn mutants into library stock plates. Knockout Sudoku includes predictive algorithms to determine how many colonies must be screened to separate multihit wells as demonstrated by the generation of the authors’ large, arrayed Shewanella oneidensis Tn library (56, 57). We did not do large-scale purification of wells with multiple mapped transposon insertions and instead focused on making smaller libraries of high-quality Tn mutants.

### SMarT TnSeq approach versus traditional TnSeq.

Most TnSeq screens employ input pools containing large numbers (∼10^5^ to 10^6^) of different mutants, with the complexity of the pools approaching saturation of all potential insertion sites in the genome outside the loci essential for viability under nonselective conditions. The use of such saturating pools containing multiple hits in nonessential loci can provide great statistical power and precision for estimating the contribution of each locus in the genome to competitive fitness under a defined condition. Many TnSeq studies, such as those focused on bacterial colonization or disease production in plant or animal hosts, examine niches with limited carrying capacities, which precludes the use of input pools with saturating diversity due to inherent bottlenecks leading to stochastic loss of mutants. While published TnSeq analyses include diverse approaches to estimating the degree of bottlenecking (22, 24, 25), there is no question that bottlenecking imposes a significant issue for many applications of TnSeq.

We began the present study with the premise that the use of sequence-defined input pools in TnSeq-based experiments might enable interrogation of the functions and fitness contributions of a large segment of the genome with input pools of lower complexity than that required for equivalent levels of coverage with pools of random mutants. The arrayed libraries used to generate these pools also facilitate immediate isolation of mutants and rapid validation of the phenotypes ascribed to individual loci based on primary screens. Approximately 150 individual mutants have been isolated from the arrayed library, verified by a secondary approach such as PCR or whole-genome sequencing, and used for additional experiments (D. Garsin, K. Kline, C. Kristich, and K. Weaver, personal communications).

In addition to decreasing issues with bottlenecking, the use of relatively small arrayed libraries has technical benefits over the use of large, random Tn pools. Our SMarT TnSeq pool contains approximately an order of magnitude fewer clones than larger random pools and yet includes insertions in a majority of genes and intergenic regions. Therefore, the number of sequencing reads required to provide sufficient coverage of Tn mutants is reduced approximately 10-fold. For example, we aimed for approximately 300 reads per Tn mutant in these experiments, which results in approximately 2.1 million reads for a SMarT TnSeq library with ∼7,000 mutants. A typical random library of approximately 70,000 mutants would require approximately 21 million reads to maintain this level of coverage. Thus, our use of SMarT TnSeq libraries enables multiplexing of more samples per sequencing lane and results in a reduction in overall sequencing costs. The data generated by sequencing smaller libraries also require less computational power for data analysis.

For many loci, more than one Tn insertion was available in the full sequenced library. When these were available, we chose to include multiple Tn mutants, which allowed us to examine the phenotypes of multiple insertions in the same gene. This can inform structural and functional genetic analysis, as disparate phenotypes for Tn mutants in the same gene may provide information regarding specific domains in gene products that are required for function under a given condition or antisense transcripts that are encoded within genes or in intergenic regions. Therefore, we believe that when multiple Tn insertions in a given gene are available for use in an arrayed library, the inclusion of *n* > 1 mutants may be beneficial.

While the SMarT TnSeq approach can be used to interrogate the fitness of a large portion of the genome with a minimal number of mutants, a downside of this approach is that it lacks the statistical power to evaluate gene essentiality. While the paucity of Tn insertions in a given genomic region may be suggestive of a locus that encodes gene products or transcripts essential for cell viability under the conditions in which our Tn library was generated, a saturating approach is needed to make high-confidence predictions regarding gene essentiality. However, we did compare the Tn insertions in our arrayed library to published data sets as a cursory evaluation of putative essential E. faecalis genes. A recent paper by Lebreton et al. (6) identified a core set of *Enterococcus* genes (*n* = 1,037) conserved across diverse isolates. Of these, 1,010 are present in OG1RF, and we found that 601 of these genes had at least 1 Tn insertion in our arrayed library. Therefore, this suggests that approximately 400 of these highly conserved *Enterococcus* genes could be potential candidates for essential genes in OG1RF.

### Arrayed transposon libraries as a general biological resource.

The use of arrayed libraries and TnSeq affords several advantages over growth experiments performed on solid medium or in microtiter plates. First, TnSeq measures the relative fitness of all mutants in a single sample, which can provide information about genetic interactions that would not be measurable in monoculture (22). Additionally, TnSeq can capture subtle phenotypes that may not be evident in alternative *in vitro* experiments. However, because the Tn mutants in the library are arrayed in individual wells, the library plates can be replica plated and used for “traditional” microtiter plate-based phenotypic screens. Small, single-plate pools can be rapidly generated for experiments with extreme biological bottlenecks, such as animal niches with a small carrying capacity. Finally, because the tubes in which the mutants are housed are marked with unique barcodes, strains can easily be reracked into libraries containing only Tn mutants in genes of interest, such as those involved in specific biological functions or pathways. Recently, a targeted Tn library containing insertions in effector genes was used to study Legionella pneumophila virulence (59). Similar arrayed Tn libraries, including those generated for Klebsiella pneumoniae (48), Burkholderia thailandensis (60), and Pseudomonas aeruginosa PAO1 (61), have enabled rapid phenotypic screens and analysis of essential genes. This report demonstrates that our libraries of arrayed E. faecalis OG1RF mutants and the SMarT TnSeq approach are significant resources for the functional genomic analysis of enterococci.

## MATERIALS AND METHODS

### Bacterial strains, antibiotics, and growth conditions.

Bacterial strains used in this study are listed in Table S4 in the supplemental material. Freezer stocks were stored at −80°C in 10 to 30% glycerol. Strains were routinely cultured in brain heart infusion (BHI) or MM9-YEG growth medium, which is a semidefined medium containing M9 salts, yeast extract, and glucose (62). For solid medium, agar was added at a final concentration of 1 to 1.5% (wt/vol) prior to autoclaving. When required for selection, the following antibiotics were used at the concentrations in parentheses: fusidic acid (Fus; 25 μg/ml), rifampin (Rif; 200 μg/ml), and erythromycin (Erm; 20 μg/ml for E. faecalis, 80 μg/ml for Escherichia coli). Gene expression from pMSP3535 derivatives was induced with the addition of nisin (50 ng/ml). Cholic acid was used at a final concentration of 0.15% (wt/vol) and was dissolved in the appropriate growth medium immediately prior to use. Antibiotics and cholic acid were purchased from Sigma. Growth medium components were purchased from Difco.

10.1128/mSystems.00062-18.10 TABLE S4 All plasmids, strains, and oligonucleotides used in this study. Download Table S4, DOCX file, 0.1 MB. Copyright © 2018 Dale et al. 2018 Dale et al. This content is distributed under the terms of the Creative Commons Attribution 4.0 International license.

### Plasmid and strain construction.

A complete list of plasmids and oligonucleotides used in this study is provided in Table S4. All PCR steps were performed using *Pfu* Ultra II Fusion HS DNA polymerase (Agilent Genomics). Restriction enzymes were purchased from New England Biolabs, and oligonucleotides were purchased from Invitrogen. DNA sequences were confirmed via Sanger sequencing (Eurofins). Plasmid propagation was performed using E. coli DH5α.

The markerless Δ*dnaK* strain was created using allelic exchange and counterselection, as described previously (63). Briefly, genomic regions upstream and downstream of *dnaK* (OG1RF_11078) were amplified using primers JD330/331 and JD332/333 and cloned into pGEM-T Easy (Promega) to create pGEM-T Easy::*dnaK*-del. The fused *dnaK* sequences were excised from pGEM-T Easy using BamHI and XmaI and further cloned into pCJK47, creating pCQP2. Deletion of *dnaK* was confirmed using primer pair JD344/JD345.

To generate the *dnaK* complementation plasmid, the *dnaK* open reading frame and ribosome binding site were amplified from E. faecalis OG1RF genomic DNA using primers JD354/JD355. The resulting PCR product was ligated to pGEM-T Easy, digested with BamHI/XmaI, and cloned into the nisin-inducible pMSP3535 vector to generate pJW174. OG1RF_10022 and OG1RF_10040 were amplified from OG1RF genomic DNA using primer pairs JW102/JW103 and JW104/JW105, respectively. PCR products were digested with BamHI-HF/SpeI and were ligated to a pMSP3535 vector prepared with the same restriction enzymes to generate plasmids pJW175 and pJW176. E. faecalis strains were made electrocompetent as described previously (64) and were transformed with pMSP3535 or the appropriate derivative. Transformants were selected on BHI-1% agar plates supplemented with Erm.

### Pooling and sequencing of the arrayed Tn library.

The library of random Tn mutants generated by Kristich et al. (18) was orthogonally pooled to facilitate sequencing of Tn site insertions. Approximately 15,000 mutants arrayed in 177 96-well microtiter plates were split into 4 sets (∼44 plates per set). All analogous columns (i.e., column 1 from each plate in the set) were combined, yielding 12 column pools. All analogous rows (i.e., row A from each plate in the set) were combined, creating 8 row pools. Finally, all wells from a single plate were combined to create single plate pools. Pooling was performed using a Rainin Liquidator 96 benchtop pipettor and 8- or 12-channel reservoir plates (Fisher Scientific).

Genomic DNA was purified using a Qiagen DNeasy kit and was submitted to the University of Minnesota Genomics Center for sequencing. DNA was sheared and processed using the Illumina TruSeq Nano library preparation kit. The libraries were normalized to 2 ng/μl, and 10 ng of each library was used as a PCR template to enrich for the *mariner* transposon junctions using a transposon-specific primer (*mariner*-seq) and the Illumina P7 primer. One microliter of the enrichment PCR product was diluted 100-fold, and 10 μl of the diluted DNA was used as the template for the indexing PCR (TruSeq P5 indexing primer + P7 primer). The final libraries had unique combinations of P5 and P7 indexes suitable for multiplexed sequencing. Libraries were quantified, normalized, and pooled. The pooled library was cleaned using 1× solid-phase reversible immobilization (SPRI) beads (AMPure XP; Beckman Coulter) and eluted in 1:10 Tris-EDTA (TE) buffer (made from 1× TE buffer purchased from Fisher). The amplicon size distribution of the pool was verified on a Bioanalyzer (Agilent), and the concentration of functional library molecules was calculated using the Kapa library quantification kit for Illumina. The pool was sequenced using an Illumina HiSeq 2500 in 100-bp paired-end output mode at the University of Minnesota Genomics Center. Plate, row, and column pools were weighted such that ∼10,000 reads were obtained for each individual well represented in a given pool. Four total lanes were used for arrayed library sequencing (1 lane per set of ∼44 96-well plates, generating approximately 1 Gb of data per plate). Reads were trimmed to remove Illumina adapter sequences using Trimmomatic and were mapped to the OG1RF reference genome (GenBank CP002621.1, NCBI RefSeq NC_017316.1) using Bowtie.

### Straight Three mapping of arrayed library mutants.

After cleanup and adapter trimming, high-quality reads associated with Tn insertion sites were traced back to freezer stock locations as follows. Noise was reduced via a series of heuristic filters applied in series. Additional detail is provided in Text S1 in the supplemental material. Data were manipulated in a relational database (FileMaker Pro) as described in the following steps. Low-abundance outliers were removed from pools where the number of mapped insertion sites exceeded the number of expected positions based on the input wells used to construct that pool. Sites where reads mapped either upstream or downstream of the Tn insertion (but not both) were removed, as Tn-chromosome junctions were amplified in a bidirectional fashion, and sequencing coverage should span the insertion site. TA sites present in >9 pools (out of the total number of pools for a given ∼44-plate set) were also discarded, as these could represent spurious reads generated by mispriming during PCR. Coefficient of variation (CV) calculations were performed to assess the relative abundance of reads associated with a TA site compared to a given pool, and sites were discarded or removed from pools if they had large variations in abundance across all pools in which they appeared. CV values were recorded and used for downstream applications, including construction of the SMarT TnSeq library.

After this data cleaning process, Tn insertion sites were assigned library plate positions. Straight Three mapping occurred when reads associated with a given TA site (i) were identified in exactly three pools (one column, one row, and one plate) and (ii) had low CV values across all pools. After assigning unambiguous Straight Three hits, a list of all possible sites for mutants appearing in exactly 6 pools (representing two sibling clones present in distinct library positions) was generated. Potential wells were discarded from this list based on known blanks and previously mapped mutants. Finally, mutants present in exactly 4 or 5 pools (representing sibling clones present with overlapping plate coordinates) were assigned to specific locations by examining the relative abundance in each pool.

### Construction of the SMarT TnSeq library and pool.

Prior to construction of the SMarT TnSeq pool, the arrayed Tn library was replica plated into individual 0.5-ml two-dimensional (2D) barcoded storage tubes (Micronic) containing 150 µl BHI-10% glycerol. The clones in the SMarT TnSeq library were then isolated using an automated XL20 tube handler (BioMicroLab) and replica plated into 96-well microtiter plates containing 150 µl BHI-10% glycerol, which were stored at −80°C. Mutants were chosen for the SMarT TnSeq library based on unique insertion sites and low coefficient-of-variation values. Mutants were excluded if library plate wells contained more than one Tn site as identified by Straight Three mapping.

A plate-scraping approach was used to generate the SMarT TnSeq pooled library. Briefly, pools were created in a stepwise manner in which 14 smaller sets of 480 to 760 Tn mutants were generated and subsequently combined into one volume of 6,829 mutants. To generate the small pools, 96-well microtiter plates containing the arrayed SMarT TnSeq library were thawed, and an 8-channel multichannel pipette was used to drop 10 µl from each well onto square petri plates (Thomas Scientific) containing BHI-1.5% agar. The plates were incubated at 37°C for 16 to 18 h. Bacteria were scraped off the plate using aseptic technique and placed into 20 ml BHI-10% glycerol. Samples were removed from each smaller pool, diluted, plated on BHI-1.5% agar to determine CFU per milliliter, and examined for contamination. Ten milliliters from each of the 14 smaller pools was combined to generate the final pool of 6,829 mutants, and an aliquot of the final pool was diluted and plated on BHI-1.5% agar to enumerate colonies and ensure that there was no contamination. Aliquots of the SMarT Tn pool were stored at −80°C.

### SMarT TnSeq growth and sampling.

To evaluate growth in cholic acid, OG1RF was grown in MM9-YEG overnight and was adjusted to a starting OD_600_ of 0.05 in fresh MM9-YEG supplemented with 0.15% (wt/vol) cholic acid or MM9-YEG alone. OD_600_ values were measured at 1-h increments. For the TnSeq experiments, frozen aliquots of the SMarT TnSeq mutant pool (input samples) were thawed on ice and used to inoculate growth media with 2.8 × 10^7^ to 4.4 × 10^7^ CFU/ml. The remaining input sample was centrifuged at 16,600 × *g*, and the pellet was stored at −80°C for DNA extractions. Cultures were incubated statically at 37°C, and samples were removed hourly to measure the OD_600_. Samples were removed for DNA extraction and sequencing at mid-exponential and late stationary phases of growth. Briefly, five 1-ml aliquots were centrifuged at 16,600 × *g*, and the pellets were stored at −80°C. Genomic DNA was extracted using the DNeasy Blood and Tissue kit (Qiagen) with minor modifications. Lysozyme (30 mg/ml; Sigma) and mutanolysin (500 U/ml; Sigma) were added to the lysis buffer to ensure sufficient lysis of E. faecalis. DNA was eluted with 100 µl of buffer AE, and the eluate was passed through the column a second time to increase DNA yields. Experiments and extractions were performed in biological duplicate. Sequencing of Tn-chromosome junctions was performed as described above with 125-bp paired-end reads, and 7 to 11 million reads were obtained for each sample.

### SMarT TnSeq analysis.

SMarT TnSeq analysis was performed using custom scripts and the Mesabi supercomputer at the Minnesota Supercomputing Institute. The technical quality of the sequencing results was evaluated using the FastQC package produced by Babraham Bioinformatics (65). We placed particular emphasis on the quality of the read tails as well as the presence of adapter contamination. The Cutadapt package was used to remove residual adapters and the primed transposon region from the sequenced forward reads (66). The trimmed reads were then aligned to the E. faecalis OG1RF reference genome using BWA-MEM (67). Mapped reads not beginning or ending at a TA site were removed due to the potential for integrations independent of transposon activity. BEDTools genomecov was used to quantify the number of integrations on a per-position basis (68). R and the package sqldf, which provides an SQL interface for R data frames (69), were used to summarize the integration data at each TA site in the OG1RF genome. These were filtered for the strains present in the SMarT TnSeq library using the 2D barcodes assigned to each transposon mutant stock tube. Integration sites, matched between sample sets, were evaluated for a significant change in the proportion of integrations received using a chi-square test and a Monte Carlo-based method that models the effect of experimental replicates and determines whether the mean proportion parameter is significantly different for each gene between experimental conditions. The chi-square results were filtered for significance (adjusted *P* < 0.05). Due to concerns about the accuracy of the chi-square approximation for some variance distribution among the replicates, a Monte Carlo approach was also applied to directly model the experimental design. This approach was highly computationally intensive and is able to estimate an E value to a minimum of only 3.73184. As a result, this value was used as an additional filter to ensure that the chi-square approximation remains sufficiently conservative. Venn diagrams were prepared with the R package VennDiagram (70).

### Microtiter plate growth curves.

A subset of transposon mutants that were underrepresented in the total E. faecalis Tn mutant population in the presence of cholic acid was selected for further analysis. Strains containing either the empty vector plasmid pMSP3535 or the appropriate pMSP3535 derivative were cultured to mid-logarithmic stage in MM9-YEG supplemented with Erm. Nisin was added for 1 h prior to starting growth curves to induce gene expression from the pMSP3535 constructs. A spectrophotometer was used to measure the OD_600_ of each culture, and cultures were adjusted to an OD_600_ of 0.05 in either MM9-YEG–Erm–nisin or MM9-YEG–Erm–nisin–0.15% cholic acid in a 96-well plate (Corning). Plates were sealed with Microseal B PCR plate sealing film (Bio-Rad). OD_600_ values were measured at 15-min intervals for 10 h using a BioTek Synergy H1 plate reader (BioTek Instruments, Inc.). Experiments were repeated in triplicate (each with two technical duplicates), and error bars show the standard error of the mean. Statistical differences between endpoint OD_600_ values were calculated using an unpaired Student *t* test with a significance cutoff of *P* < 0.05.

### Data availability.

The unprocessed FASTQ files generated from TnSeq have been deposited with the NCBI Sequence Read Archive under SRA accession number SRP150800. The custom scripts used for TnSeq analysis can be accessed at https://github.com/dunnylabumn/Ef_OG1RF_tnseq.
