# Supplementary material for: Comprehensive Functional Analysis of the Enterococcus faecalis Core Genome Using an Ordered, Sequence-Defined Collection of Insertional Mutations in Strain OG1RF
Source: mSystems. 2018 Sep 11;3(5):e00062-18. doi: 10.1128/mSystems.00062-18 (PMC6134198; doi:10.1128/mSystems.00062-18)
Supplement: FIG S1 [file sys004182258sf1.pdf]

A) *Sibling clones – same plate*

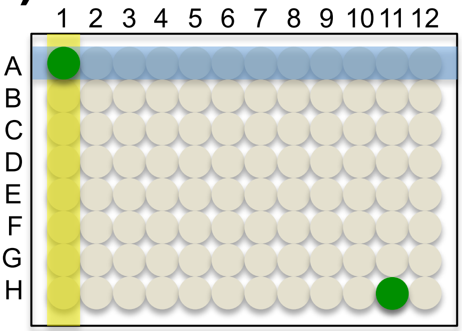

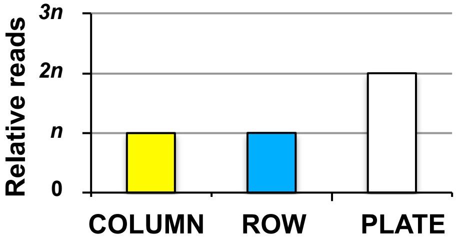

COLUMN 1 Plate	COLUMN 1 Well	ROW A Plate	ROW A Well	PLATE 1 Plate	PLATE 1 Well
**1**	**A1**	**1**	**A1**	**1**	**A1**
1	B1	1	A2	1	A2
1	C1	1	A3	1	A3
…	…	…	…	1	A4
2	A1	2	A1	1	A5
2	B1	2	A2	…	...
2	C1	2	A3	1	H8
...	...	...	...	1	H9
44	A1	44	A1	1	H10
44	B1	44	A2	**1**	**H11**
44	C1	44	A3	1	H12

B) *Multiple Tn insertions in a single well*

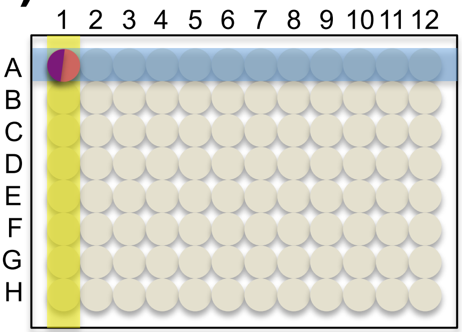

INSERTION 1
ATCGATTCG**TA**ATCTATCG

INSERTION 2
GCATCTATG**TA**GCAGCTAT

COLUMN 1 Plate	COLUMN 1 Well	ROW A Plate	ROW A Well	PLATE 1 Plate	PLATE 1 Well
**1**	**A1**	**1**	**A1**	**1**	**A1**
1	B1	1	A2	1	A2
**1**	**A1**	**1**	**A3**	1	A3
…	…	…	…	1	A4
2	A1	2	A1	**1**	**A1**
2	B1	2	A2	…	...
2	C1	2	A3	1	H8
...	...	...	...	1	H9
44	A1	44	A1	1	H10
44	B1	44	A2	1	H11
44	C1	44	A3	1	H12
