# Supplementary material for: Comprehensive Functional Analysis of the Enterococcus faecalis Core Genome Using an Ordered, Sequence-Defined Collection of Insertional Mutations in Strain OG1RF
Source: mSystems. 2018 Sep 11;3(5):e00062-18. doi: 10.1128/mSystems.00062-18 (PMC6134198; doi:10.1128/mSystems.00062-18)
Supplement: TEXT S1 [file sys004182258s1.pdf]

The mapping of genomic loci observed in raw NGS data from plate, row, and column pools involved a sequential series of filtering steps. The raw NGS data is noisy, **with *bona fide* "signals"** (loci that represent true insertional mutants amplified from genomic DNA libraries via Mariner-specific sequences) contaminated by both **low-level noise** (random fragments of gDNA libraries that map to potential insertional sites) and **high-level noise** (off-target amplification of certain loci that, for example, are generated by non-specific amplification using Mariner-specific primers). Noise was reduced via a series of heuristic filters applied in series, as described. Data was manipulated in a relational database (FileMaker Pro) as described in the following steps.

**STEP 1: PRIMARY DATA FILTERING**

1) For each record containing data with following raw information:
   - POOL_ID
   - LOCUS
   - ORIENTATION (+ or - STRAND)
   - READ COUNT (# READS)
2) Discard all POOL_ID-LOCUS combinations for which there are not at least 1 read detected on *both* strands (+ and - strand)
3) Discard all LOCI combinations that are not found in at least 1 ROW POOL-LOCUS, 1 COLUMN POOL-LOCUS, and 1 PLATE POOL-LOCUS

   *Rationale of heuristic:*

a) True genomic loci from actual Mariner-containing amplified loci will be detected on both strands due to amplification from both ends of Mariner insertion, whereas background sequencing noise from unamplified genomic loci may not.
b) Loci that do not appear in at least one of each type of pool cannot be successfully mapped to a well.

Data that survives STEP 1 progresses to STEP 2.

**STEP 2: POOL-LEVEL FILTERING**

For *each* pool (each row, column, and plate pool), *independently*:

1) Calculate the normalized pool abundance (NPA) of each locus *L* within pool as (READ COUNT of locus *L*) / (sum of READ COUNT of all loci in pool), such that the mean NPA = 1.0
2) Sort all loci in pool from highest to lowest NPA
3) Filter the number of loci by NPA (discard loci with NPA ≤ 0.01, or 1% of the mean NPA)

*Rationale of heuristic:*

a) Genomic loci detected from background sequencing noise are significantly less abundant than *bona fide* signal from actual Mariner-containing amplified loci.
b) Sensitivity threshold: we desire to map a hypothetical poorly growing mutant at as low as 1% of mean NPA.
c) Due to imbalance between the total read numbers generated for different pools in an NGS sequencing lane, normalizing *within* a pool generates a relative normalized abundance measurement that is comparable *between* pools for a given *locus*, as it mostly reflects the relative abundance of an isolate in the original culture well used in pool creation. This relative measurement is used in LOCUS-BASED FILTERING in STEP 3.

*Illustration of heuristic (see figure, below)*. LEFT: all loci for a column pool from 38 plates are shown on the left, in terms of raw read counts sorted from most abundant to least abundant locus from left to right. Expected number of loci was 38 columns x 8 wells/column – 7 blank wells = 297 loci. Observed number of loci was 486. RIGHT: filtered loci for a column pool of 38 plates are show on the right, in terms of NPA sorted from most abundant to least abundant locus from left to right. After removal of loci at > 0.1% of mean NPA, 292 loci remain.

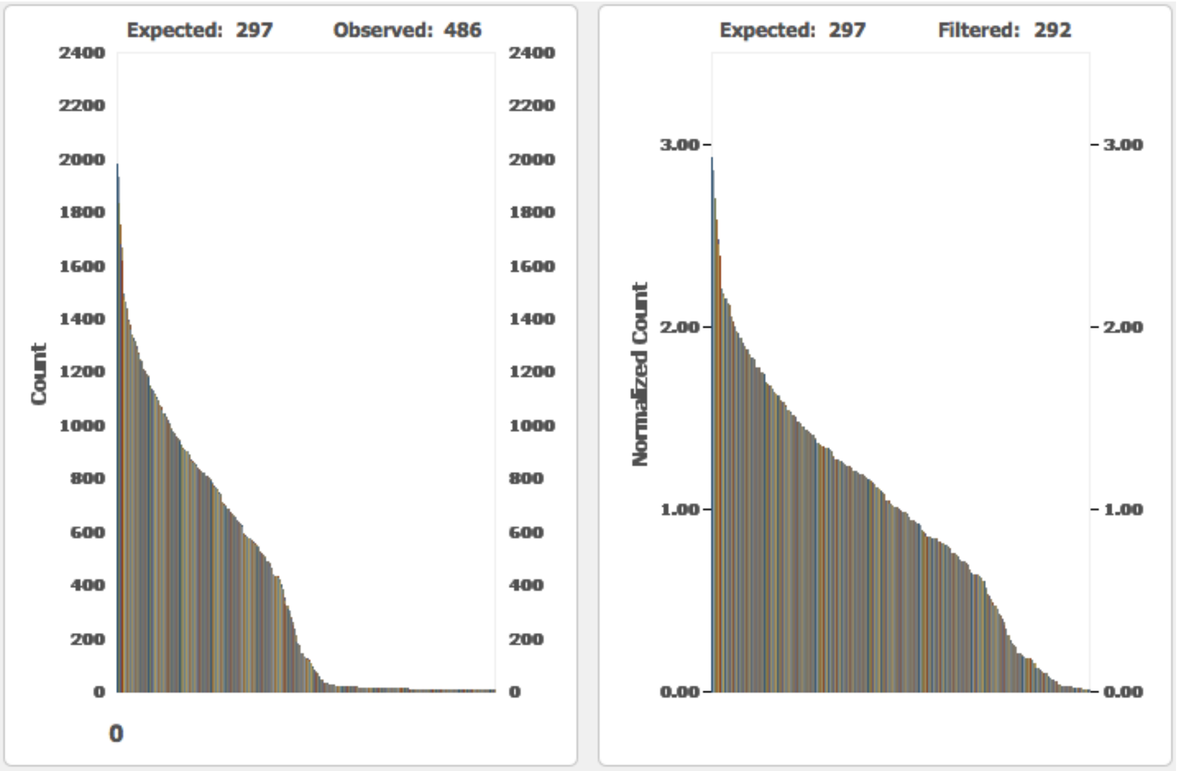


Data that survives STEP 2 progresses to STEP 3.

**STEP 3: LOCUS-LEVEL FILTERING**

In theory, a locus *may* be observed in any number of pools (between 1 and all pools). Ideally, a locus is found in exactly three pools (1 ROW, 1 COLUMN, 1 PLATE), and indeed, the majority of loci (≈ 60%) passed to STEP 3 were found in precisely three pools. However, due to the noisiness of raw NGS data in this approach – even after POOL-LEVEL PARSING – many loci were found in *more than* three pools.

The presence of a *bone fide* locus signal in 4, 5, 6 or more pools is possible, for example, when a Mariner insertion in the locus occurs in more than one clone in the population being mapped. Hence, a small number of loci, either due to such representation in multiple wells *or* due to high-level noise from non-specific amplification of genomic loci, appeared in a *large* number of pools. In order to remove noise from these data, then, while retaining true signals of loci that map to > 1 well and truly exist in > 3 pools, an iterative filtering heuristic was applied to each locus, as follows.

For *each* locus *independently*,

1) Sort locus pools from most-to-least *abundant* by NPA
2) Calculate the **normalized locus abundance (NLA)** of pools, by calculating the mean NPA of the pools, and dividing each pool's NPA by the mean NPA of all pools for the locus
3) Examine the least abundant pool for the locus, and determine if its **NPA < 0.05** and its **NLA is < 0.1**.
4) If the outcome of 3) is YES, discard the data and iterate from 2). If the outcome of 3) is NO, then STOP.

*Rationale of heuristic:*

a) For a given clone, its relative normalized abundance will be relatively constant across pools, hence a dense clonal outgrowth well and/or an insertional position that amplifies effectively will be well represented in all pools in which it is found, whereas a low-titer clonal outgrowth well and/or an insertion that amplifies poorly will be poorly represented in all pools in which it is found
b) Background NGS noise will be found at much lower abundance than *bona fide* signal
c) Hence, filtering pools that are abundance outliers will eliminate noise while retaining bone fide signal

*Illustration of heuristic (see figures below)*. TOP: an example of a locus found in 7 pools (2 plate ("rack") pools, 3 row pools, and 2 column pools). BOTTOM: Heuristic filtering as described above was applied, resulting in six pools. The fact that the remaining number of pools includes equal numbers (2) of rack, row, and column pools indicates that this locus is likely found in two wells. The fact that two discrete clusters of NPA and NLA exist also suggests that the best possible mappings of two clones is Rack 105 G01 and Rack 065 A05.

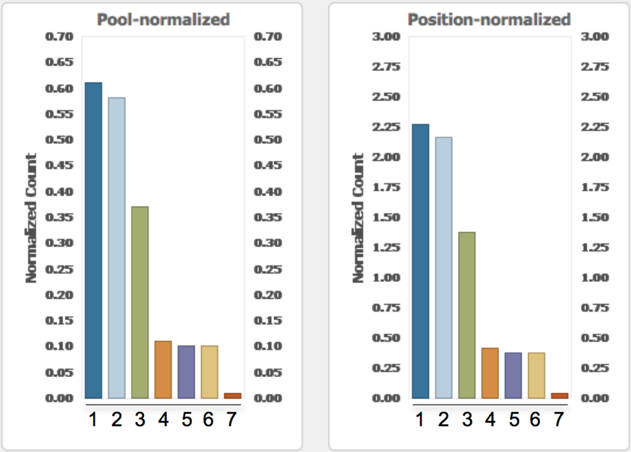


| | Pool | Pool-Normalized | Position-Normalized | 0/1 |
|---|---|---|---|---|
| 1 | Row_G | 0.61 | 2.27 | 1 |
| 2 | Column_01 | 0.58 | 2.16 | 1 |
| 3 | Rack_105 | 0.37 | 1.38 | 1 |
| 4 | Row_A | 0.11 | 0.41 | 1 |
| 5 | Column_05 | 0.10 | 0.37 | 1 |
| 6 | Rack_065 | 0.10 | 0.37 | 1 |
| 7 | Row_E | 0.01 | 0.04 | 1 |

DATA RECORDS (ON) — Racks 2 | Rows 3 | Cols 2 | 0.27 | CV 93%

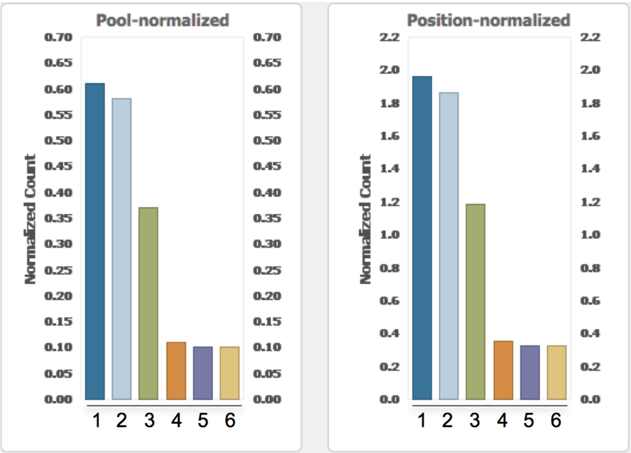


| | Pool | Pool-Normalized | Position-Normalized | 0/1 |
|---|---|---|---|---|
| 1 | Row_G | 0.61 | 1.96 | 1 |
| 2 | Column_01 | 0.58 | 1.86 | 1 |
| 3 | Rack_105 | 0.37 | 1.19 | 1 |
| 4 | Row_A | 0.11 | 0.35 | 1 |
| 5 | Column_05 | 0.10 | 0.32 | 1 |
| 6 | Rack_065 | 0.10 | 0.32 | 1 |

DATA RECORDS (ON) — Racks 2 | Rows 2 | Cols 2 | 0.31 | CV 78%

*Illustration of heuristic (see figures below)*. LEFT: The distribution of pools/locus across all loci before STEP 3; roughly 60% of loci are found in 3 pools. RIGHT: Heuristic LOCUS-LEVEL filtering results in an increase in the number of loci found in exactly three pools to ≈ 85%, and reduces dramatically the number of loci associated with large numbers of pools.

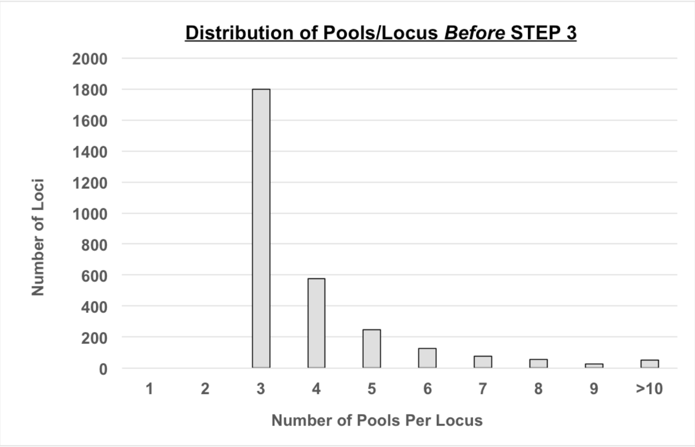


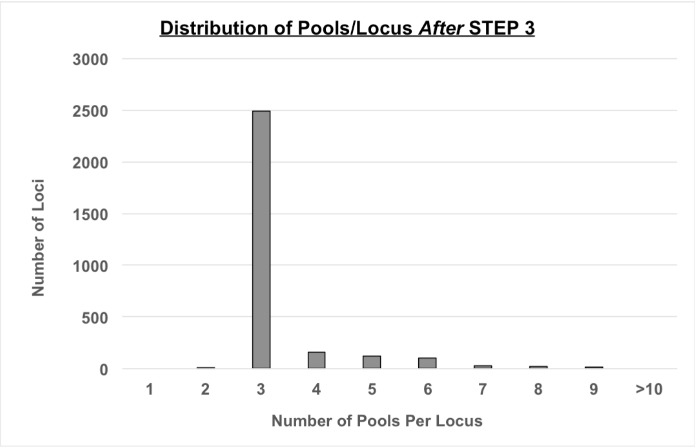

**STEP 4: GENERATION OF ALL POSSIBLE MAPPINGS**

After heuristic filtering of background noise in STEPS 1 – 3, the remaining data were used to generate all possible mappings of loci to wells. For example, a locus found in exactly 1 plate, 1 row, and 1 column pool generates one possible mapping to one well. We refer to such wells as "STRAIGHT-THREE" wells, as there is no branching in the combinatorial graph for assigning such a locus to a well.

A locus found in exactly 4 pools (e.g., 1 plate, 1 rows, 2 columns), on the other hand, generates two possible well mappings to the same plate and row, but two different columns. A locus found in exactly 6 pools, for example, (2 plates, 2 rows, 2 columns) generates eight possible mappings. With seven or more pools for a locus, the number of possible mappings grows unmanageable. As there were few loci that were found in ≥ 7 pools, and as it was also deemed likely that such residual loci were possible due to non-specific amplification (high-level noise), all such loci were discarded in this step.

In creating potential mappings, a coefficient of variance for relative abundance was calculated for each possible set of pools using the normalized locus abundance described above. For example, referring to the figure above in STEP 3, the CV for mapping Rack 105 G01 is the CV of (1.96, 1.86, 1.19) and the CV for mapping Rack 065 A05 is the CV of (0.35, 0.32, 0.32), whereas the CV of mapping of Rack 065 G01 is the CV of (1.96, 1.86, 0.32). This CV is a metric used in heuristic scoring in STEP 5, where lower = better.

**STEP 5: HEURISTIC SELECTION AND SCORING OF POSSIBLE MAPPINGS**

The final step of mapping involves assigning possible mappings to wells. This involves an initial round of conservative mapping, followed by a round of semi-conservative mapping.

**Conservative mapping.** Loci are mapped to wells when the well is associated with a single proposed locus and that mapping proposal has a CVs of relative abundance between its pools < 100%. For **conservative** STRAIGHT-THREE mappings, a grade of A+ is assigned as there is no ambiguity in the assignment. Following mapping of conservative STRAIGHT THREE wells, conservative mapping of loci containing 6, 5, and then 4 pools follow, choosing between multiple alternative pairs of mappings through the use of three heuristics: a) parsimony (e.g., for a locus with 6 pools, each pool is mapped only once to a well), b) complementarity (e.g., for a locus with 6 pools, the four complementary pairs of mappings are considered as pairs, and BOTH must be conservatively mapped), and c) score (in cases where there is more than one possible pair of complementary conservative mappings, the lowest CVs of relative abundance are used to assign a winning pair).

**Semi-conservative mapping.** Finally**,** semi-conservative mapping is carried out, again starting with 3-pool loci followed by loci with 6, 5, and 4 pools. Here, mapping of a relatively small residual number of loci is achieved into wells that may ultimately wind up with more than a single mapped locus. The same heuristics described for conservative mapping are used to choose between pairs of mappings for loci with > 3 pools.
