# Supplementary figures and images for: Comprehensive Functional Analysis of the Enterococcus faecalis Core Genome Using an Ordered, Sequence-Defined Collection of Insertional Mutations in Strain OG1RF
Source: mSystems. 2018 Sep 11;3(5):e00062-18. doi: 10.1128/mSystems.00062-18 (PMC6134198; doi:10.1128/mSystems.00062-18)

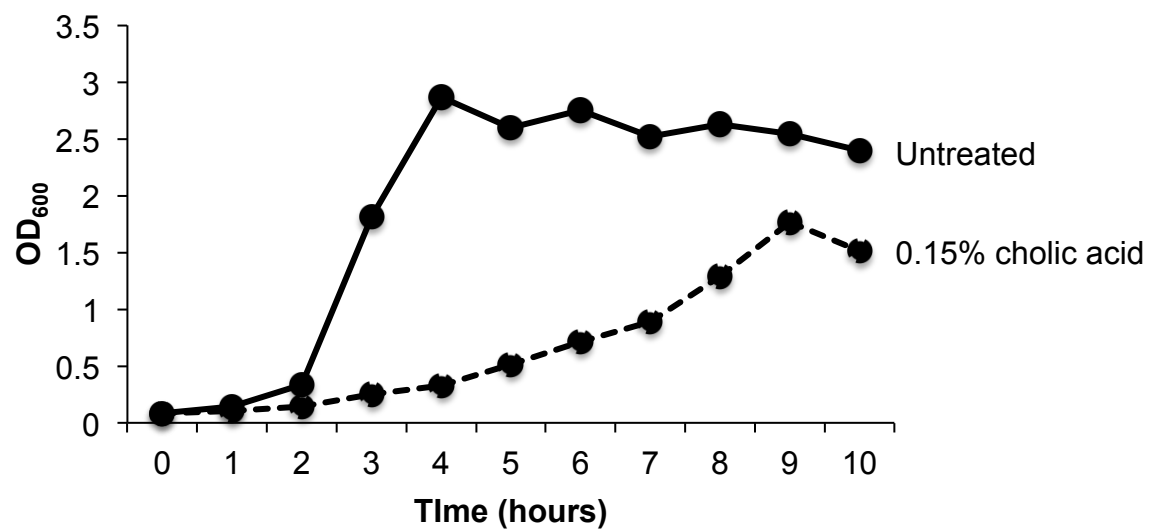

Supplement: FIG S2 [file sys004182258sf2.pdf]

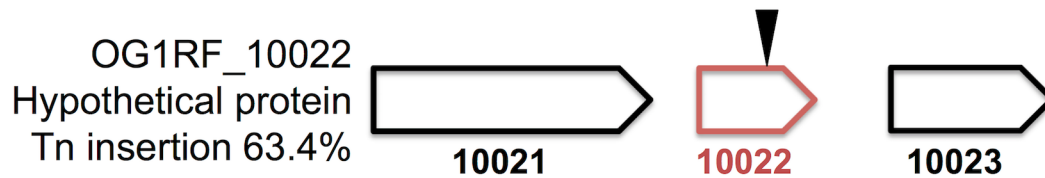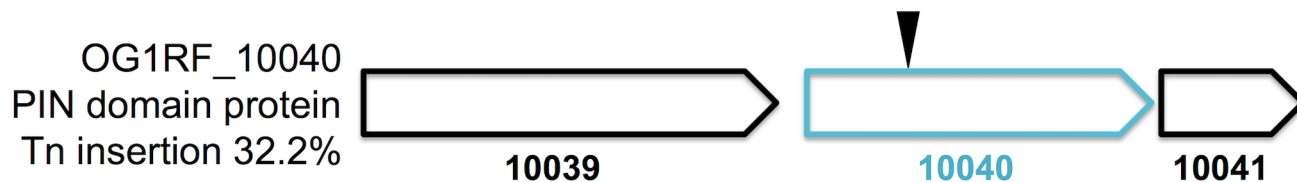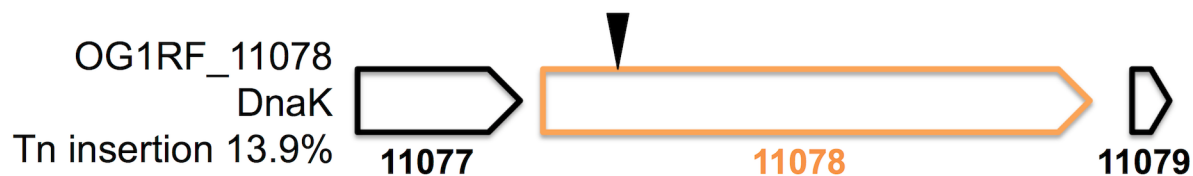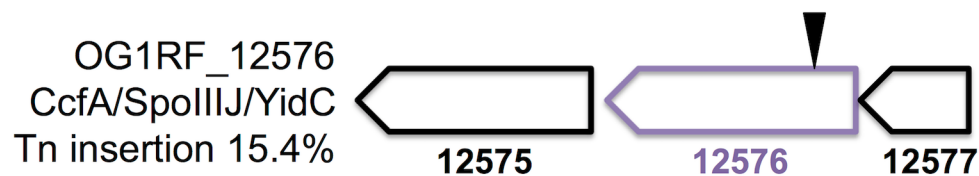

Supplement: FIG S3 [file sys004182258sf3.pdf]
