# Supplementary material for: Comprehensive Functional Analysis of the Enterococcus faecalis Core Genome Using an Ordered, Sequence-Defined Collection of Insertional Mutations in Strain OG1RF
Source: mSystems. 2018 Sep 11;3(5):e00062-18. doi: 10.1128/mSystems.00062-18 (PMC6134198; doi:10.1128/mSystems.00062-18)
Supplement: TABLE S4 [file sys004182258st4.docx]

| ***Strains*** | ***Relevant Characteristics*** | ***Source*** |
| --- | --- | --- |
| *Enterococcus faecalis* OG1RF | Parent strain, Rif^R^, Fus^R^ | (1) |
| *Enterococcus faecalis* OG1RF *ccfA* LAT | Chromosomal point mutation in *ccfA* (OG1RF_12576) (MKKYKRLLMAGL(V🡪A)TLVFV), results in non-functional cCF10 pheromone but does not disrupt translation of mature CcfA | (2) |
| *Enterococcus faecalis* OG1RF *ccfA-2* | Chromosomal stop codons (MKKYKRLLMAG**), strain does not produce cCF10 or mature CcfA | (2) |
| *Enterococcus faecalis* OG1RF Δ*dnaK* | Markerless deletion of *dnaK* (OG1RF_11078) | This study |
| *Escherichia coli* DH5α | Laboratory K-12 cloning strain | Fisher Scientific |
|  |  |  |
| ***Plasmids*** | ***Relevant Characteristics*** | ***Source*** |
| pCJK47 | Conjugative donor plasmid, carries *oriT*_pCF10_and P-*pheS**, pORI280 derivative, Erm^R^ | (3) |
| pCQP2 (pCJK47::*dnaK*-del) | Conjugative donor plasmid containing sequence upstream and downstream of *dnaK* (OG1RF_11078), Erm^R^ | This study |
| pGEM-T Easy | *lacZ*, cloning vector, Amp^R^ | Promega |
| pGEM-T Easy::*dnaK*-del | Contains sequence upstream and downstream of *dnaK* (OG1RF_11078), Amp^R^ | This study |
| pMSP3535 | Nisin-inducible gene expression, Erm^R^ | (4) |
| pMSP3535::*ccfA* | Contains wild-type *ccfA* sequence, Erm^R^ | (5) |
| pJW174 (pMSP3535::*dnaK*) | Contains wild-type *dnaK* sequence, Erm^R^ | This study |
| pJW175 (pMSP3535::OG1RF_10022) | Contains wild-type OG1RF_10022 sequence, Erm^R^ | This study |
| pJW176 (pMSP3535::OG1RF_10040) | Contains wild-type OG1RF_10040 sequence, Erm^R^ | This study |
|  |  |  |
| ***Oligonucleotides*** | ***Sequence*** | ***Source*** |
| 10022-Bam-fwd | 5' – AAT GGA TCC AAT GAA ATG GAG CAG ATA ACA TGG - 3' | This study |
| 10022-Spe-rev | 5' – TTT ACT AGT AAC CGC TAC TTT TTC ATC TAG - 3' | This study |
| 10040-Bam-fwd | 5' – ATA GGA TCC ATT AAA TAA ACG AGG AGG ATG GC - 3' | This study |
| 10040-Spe-rev | 5' – AAT CAT ACT AGT CCT CAC TTT GC - 3' | This study |
| JD330 (BamHI) | 5’ – GGA TCC CTT TAT GCA AAA TTC TGA TGA GCT G – 3’ | This study |
| JD331 | 5’ – AAT GAA GAA GTT TTT GTT GTT CCT AAG TCA ATA CCA AT – 3’ | This study |
| JD332 | 5’ – AAT CCT TGT TGT TTT TGA AGA AGT AAA TGG TGA TGA CA – 3’ | This study |
| JD333 (XmaI) | 5’ – CCC GGG AGT ATG TGC TTT TTT CTC ATG ACC – 3’ | This study |
| JD354s (BamHI) | 5’ – GGA TCC CAG ATT GGA GAT ATT CAC TAT G – 3’ | This study |
| JD355as (XmaI) | 5’ – CCC GGG TTA TTT GTC ATC ACC ATT TAC TTC – 3’ | This study |
| *mariner*-seq | 5’ – TCG TCG GCA GCG TCA GAT GTG TAT AAG AGA CAG CCG GGG ACT TAT CAG CCA ACC – 3’ | This study |

**Table S4. Bacterial strains, plasmids, and oligonucleotides used in this study.** Amp^R^, ampicillin; Erm^R^, erythromycin; Fus^R^, fusidic acid; Rif^R^, rifampicin. Restriction enzyme sites in oligonucleotides are included in the name or in parentheses. The underlined nucleotides in *mariner*-seq anneal to the *mariner* transposon.

1. Dunny G, Funk C, Adsit J. Direct stimulation of the transfer of antibiotic resistance by sex pheromones in Streptococcus faecalis. Plasmid. 1981;6(3):270-8. PubMed PMID: 6796985.

2. Chandler JR, Hirt H, Dunny GM. A paracrine peptide sex pheromone also acts as an autocrine signal to induce plasmid transfer and virulence factor expression in vivo. Proc Natl Acad Sci U S A. 2005;102(43):15617-22. Epub 2005/10/13. doi: 10.1073/pnas.0505545102. PubMed PMID: 16223881; PubMed Central PMCID: PMCPMC1266105.

3. Kristich CJ, Chandler JR, Dunny GM. Development of a host-genotype-independent counterselectable marker and a high-frequency conjugative delivery system and their use in genetic analysis of Enterococcus faecalis. Plasmid. 2007;57(2):131-44. doi: 10.1016/j.plasmid.2006.08.003. PubMed PMID: 16996131; PubMed Central PMCID: PMCPMC1852458.

4. Bryan EM, Bae T, Kleerebezem M, Dunny GM. Improved vectors for nisin-controlled expression in gram-positive bacteria. Plasmid. 2000;44(2):183-90. doi: 10.1006/plas.2000.1484. PubMed PMID: 10964628.

5. Antiporta MH, Dunny GM. ccfA, the genetic determinant for the cCF10 peptide pheromone in Enterococcus faecalis OG1RF. J Bacteriol. 2002;184(4):1155-62. PubMed PMID: 11807076; PubMed Central PMCID: PMCPMC134800.
